# Autophagy promotes cell survival by maintaining NAD levels

**DOI:** 10.1016/j.devcel.2022.10.008

**Published:** 2022-11-21

**Authors:** Tetsushi Kataura, Lucia Sedlackova, Elsje G. Otten, Ruchika Kumari, David Shapira, Filippo Scialo, Rhoda Stefanatos, Kei-ichi Ishikawa, George Kelly, Elena Seranova, Congxin Sun, Dorothea Maetzel, Niall Kenneth, Sergey Trushin, Tong Zhang, Eugenia Trushina, Charles C. Bascom, Ryan Tasseff, Robert J. Isfort, John E. Oblong, Satomi Miwa, Michael Lazarou, Rudolf Jaenisch, Masaya Imoto, Shinji Saiki, Manolis Papamichos-Chronakis, Ravi Manjithaya, Oliver D.K. Maddocks, Alberto Sanz, Sovan Sarkar, Viktor I. Korolchuk

**Affiliations:** 1 Biosciences Institute, Faculty of Medical Sciences, Newcastle University, Newcastle upon Tyne NE4 5PL, UK; 2 Department of Biosciences and Informatics, Keio University, Yokohama, Kanagawa 223-8522, Japan; 3 Department of Neurology, Juntendo University School of Medicine, Bunkyo, Tokyo 113-8421, Japan; 4 Autophagy lab, Molecular Biology and Genetics Unit, Jawaharlal Nehru Centre for Advanced Scientific Research (JNCASR), Jakkur, Bangalore 560064, India; 5 Wellcome Centre for Mitochondrial Research, Newcastle University, Newcastle upon Tyne, UK; 6 Center for Genomic and Regenerative Medicine, Juntendo University Graduate School of Medicine, Bunkyo, Tokyo 113-8421, Japan; 7 Institute of Cancer and Genomic Sciences, Institute of Biomedical Research, College of Medical and Dental Sciences, University of Birmingham, Birmingham B15 2TT, UK; 8 Whitehead Institute for Biomedical Research, Massachusetts Institute of Technology, Cambridge, MA 02142, USA; 9 Institute of Systems, Molecular and Integrative Biology, University of Liverpool, Liverpool L69 7BE, UK; 10 Department of Neurology, Mayo Clinic, 200 First St. SW, Rochester, MN 55905, USA; 11 Institute of Cancer Sciences, University of Glasgow, Glasgow G61 1QH, UK; 12 Department of Molecular Pharmacology and Experimental Therapeutics, Mayo Clinic, 200 First St. SW, Rochester, MN 55905, USA; 13 The Procter & Gamble Company, Cincinnati, OH 45040, USA; 14 Department of Biochemistry and Molecular Biology, Biomedicine Discovery Institute, Monash University, Melbourne, VIC 3800, Australia; 15 Walter and Eliza Hall Institute of Medical Research, Parkville, VIC, Australia; 16 Department of Biology, Massachusetts Institute of Technology, Cambridge, MA 02142, USA; 17 Division for Development of Autophagy Modulating Drugs, Juntendo University Graduate School of Medicine, Bunkyo, Tokyo 113-8421, Japan; 18 School of Molecular Biosciences, College of Medical, Veterinary and Life Sciences, University of Glasgow, Glasgow G12 8QQ, UK; 19 Novartis Institutes for Biomedical Research, Shanghai, China; 20 Present address: Centre for Genomic Regulation (CRG), The Barcelona Institute of Science and Technology, Barcelona, Spain; 21 Present address: MRC Laboratory for Molecular Biology, Cambridge, UK; 22 Present address: Università degli Studi della Campania “Luigi Vanvitelli”, Caserta, Italy; 23 Present address: Triphase Accelerator, Toronto, ON, Canada; 24 These authors contributed equally; 25 Lead contact

## Abstract

Autophagy is an essential catabolic process that promotes the clearance of surplus or damaged intracellular components. Loss of autophagy in age-related human pathologies contributes to tissue degeneration through a poorly understood mechanism. Here, we identify an evolutionarily conserved role of autophagy from yeast to humans in the preservation of nicotinamide adenine dinucleotide (NAD) levels, which are critical for cell survival. In respiring mouse fibroblasts with autophagy deficiency, loss of mitochondrial quality control was found to trigger hyperactivation of stress responses mediated by NADases of PARP and Sirtuin families. Uncontrolled depletion of the NAD(H) pool by these enzymes ultimately contributed to mitochondrial membrane depolarization and cell death. Pharmacological and genetic interventions targeting several key elements of this cascade improved the survival of autophagy-deficient yeast, mouse fibroblasts, and human neurons. Our study provides a mechanistic link between autophagy and NAD metabolism and identifies targets for interventions in human diseases associated with autophagic, lysosomal, and mitochondrial dysfunction.

## In brief

Kataura and Sedlackova et al. demonstrate an evolutionarily conserved role of autophagy, and specifically mitophagy, in the preservation of nicotinamide adenine dinucleotide (NAD) levels and cell survival. Viability of autophagy-deficient cells was improved by pharmacological interventions targeting NAD metabolism, providing insights for the treatment of human diseases associated with autophagic and mitochondrial dysfunction.

## Graphical Abstract

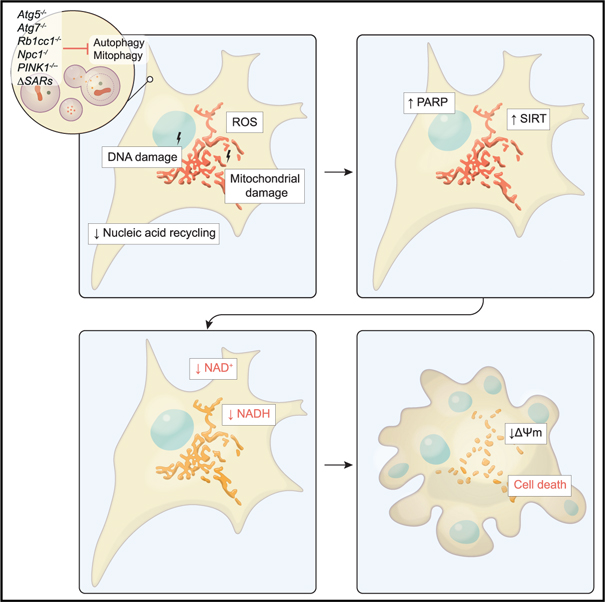

## INTRODUCTION

Autophagy is a cellular trafficking pathway mediated by the formation of double-membraned vesicles called autophagosomes, which ultimately fuse with lysosomes where their cargo is degraded. By sequestering and clearing dysfunctional cellular components, such as protein aggregates and damaged organelles, autophagy maintains cellular homeostasis, while also providing metabolites and energy during periods of starvation.^1^ Studies using a range of laboratory models from yeast to mammals have established that autophagy is essential for cellular and organismal survival. For example, loss of an essential autophagy gene *atg5* leads to reduced survival of *Saccharomyces cerevisiae* in nitrogen starvation conditions and shortened lifespan in *Drosophila melanogaster*.^2,3^ Likewise, inducible knockout of *Atg5* results in cell death and neurodegeneration in adult mice.^4–6^ Impairment of autophagy has also been implicated in various human pathologies such as lysosomal storage disorders and neurodegenerative diseases.^7^ However, it remains unclear which of the many physiological functions of autophagy are most important for its role in maintaining cell and organismal survival. Furthermore, the role of autophagy in the quality control of cellular proteins and organelles is likely to impact a plethora of signal transduction and stress response pathways, which in turn affect metabolism, growth, and survival.^8,9^ Untangling this complexity *in vivo* is challenging, although mechanistic studies of the cellular roles of autophagy *in vitro* are hindered by the fact that autophagy-deficient cells are viable in cell culture.^5,6,10^ We hypothesized that this apparent discrepancy between the requirement for functional autophagy *in vivo* and *in vitro* could be due to a metabolic shift from oxidative phosphorylation (OXPHOS) to glycolysis in tissue culture, which could mask an underlying viability defect in autophagy-deficient cells.^11^

## RESULTS

### Cell death underlying loss of autophagy is associated with NAD(H) depletion

**Figure 1.**
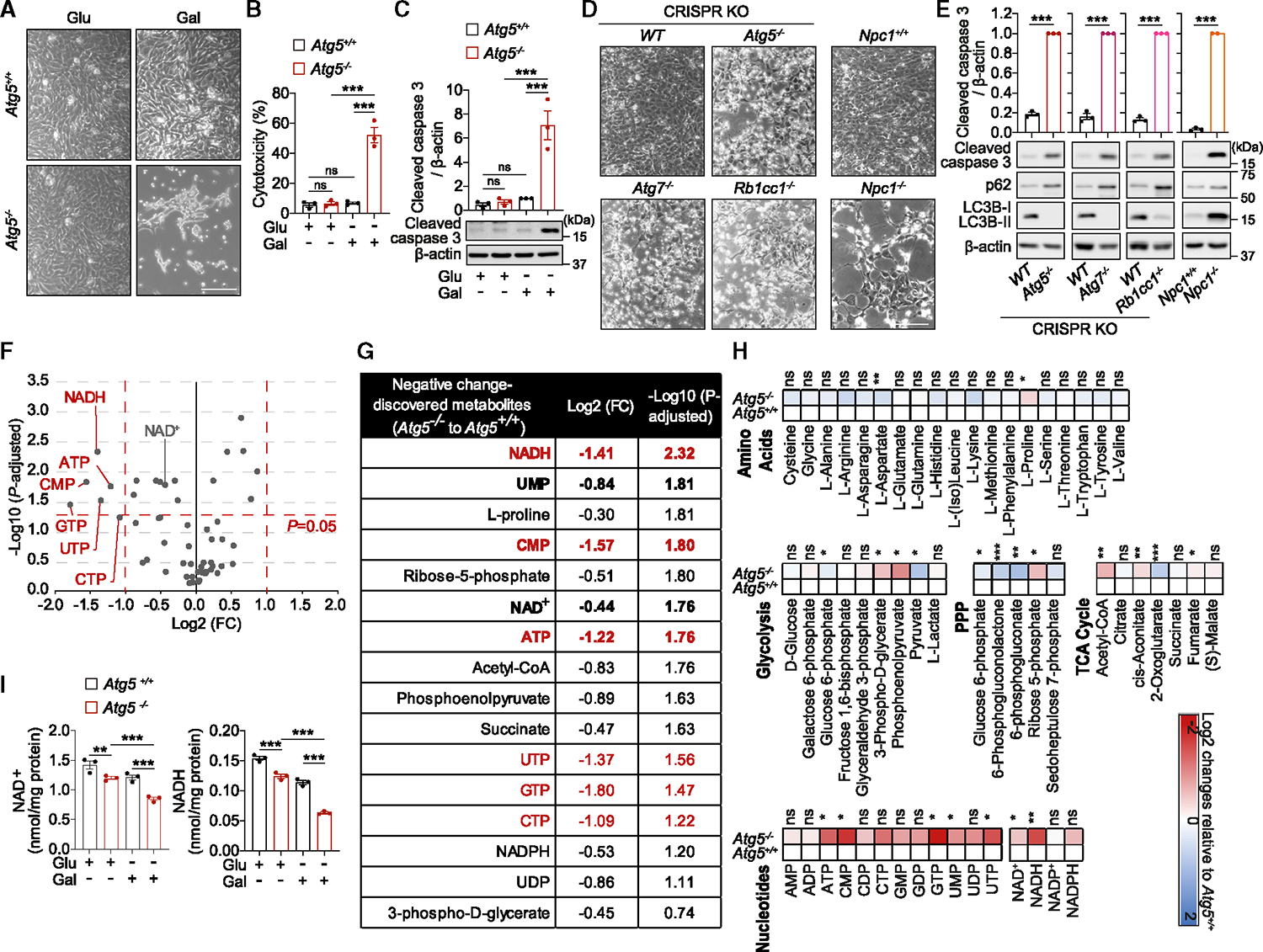
Metabolic deficit and apoptotic cell death in respiring autophagy-deficient cells (A–C) Apoptotic cell death in respiring *Atg5*^−/−^ MEFs. Phase contrast images (A), cytotoxicity assays (B), and immunoblot analyses for caspase-3 cleavage (C) of *Atg5*^+/+^ or *Atg5*^−/−^ MEFs cultured in glucose (glu) or galactose (gal) medium for 24 h (A and C) or 40 h (B). (D and E) Apoptotic cell death in respiring cells with the deletion of autophagy-related genes. Phase contrast images (D) and immunoblotting analyses for caspase-3 cleavage (E) of wild-type (*WT*) or isogenic *Atg5*^−/−^, *Atg7*^−/−^, and *Rbcc1*^−/−^ cell lines generated by the CRISPR-Cas9 system; and *Npc1*^+/+^ and *Npc1*^−/−^ MEFs grown in gal medium for 110 h (*Atg5*, *Atg7*, and *Rb1cc1 CRISPR*-Cas9 generated cell lines) or 72 h (*Npc1* cell lines). (F) Volcano plot representation of all analyzed metabolites in a pairwise comparison of *Atg5*^−/−^ to *Atg5*^+/+^ MEFs after 16 h in gal medium. Thresholds are shown as dashed red lines. (G) List of discovered depleted metabolites in *Atg5*^−/−^ MEFs. Highlighted are metabolites that change significantly (−1 ≥ log_2_(FC) ≥ 1, log_10_[p adjusted > 1.3, highlighted in red]) and correlate with cell death/survival (bold). (H) Metabolite profiling in *Atg5*^+/+^ and *Atg5*^−/−^ MEFs is depicted as a heatmap of log_2_(FC) of *Atg5*^−/−^ to *Atg5*^+/+^ MEFs based on their association to glucose oxidation pathways of glycolysis, pentose phosphate pathway (PPP), and tricarboxylic acid (TCA) cycle. (I) NAD(H) pool is depleted in respiring *Atg5*^−/−^ MEFs. Measurement of NAD^+^ and NADH levels in *Atg5*^+/+^ and *Atg5*^−/−^ MEFs cultured in glu or gal medium for 20 h. Graphical data are mean ± SEM of n = 3 biological replicates (B, C, E, and I). p values were calculated by one-way ANOVA followed by multiple comparisons with the two-stage linear step-up procedure of Benjamini, Krieger, and Yekutieli (B, C, and I), unpaired two-tailed Student’s t test (E) or multiple t test with the original FDR method of Benjamini and Hochberg (F and H) on three independent experiments. *p < 0.05; **p < 0.01; ***p < 0.001; ns (non-significant). Scale bars, 200 μm in (A) and (D). See also Figure S1.

A well-established strategy to reverse cellular reliance on energy generation via aerobic glycolysis and promote mitochondrial OXPHOS *in vitro* is to replace glucose, the major carbon source in tissue culture media, with galactose.^12–16^ Strikingly, *Atg5*^−/−^ but not wild-type (WT) mouse embryonic fibroblasts (MEFs) cultured in galactose media displayed rapid (~24 h) caspase activation and cell death (Figures 1A–1C). This phenotype was not caused by galactose directly, as it was not toxic in the presence of glucose (Figures S1A and S1B). Suppressing mitochondrial respiration via hypoxia or by UK-5099, an inhibitor of pyruvate (mitochondrial complex I [CI] substrate) carrier into mitochondria, rescued cell viability (Figures S1C–S1F). Conversely, increased concentrations of pyruvate fueling mitochondrial respiration exacerbated cell death (Figures S1G and S1H). Together, these observations implicated the role of respiring mitochondrial in the demise of autophagy-deficient cells. Specificity of this phenotype was validated by the re-expression of Atg5, and the apoptotic nature of cell death as evidenced by the caspase-3 cleavage was confirmed using Z-VAD-fmk (Figures S1I–S1K). Apoptosis upon culture in galactose medium was also observed in cell lines with CRISPR-Cas9 knockout of key autophagy genes *Atg5*, *Atg7*, or *Rb1cc1* (homolog of human *FIP200*), as well as with the loss of lysosomal cholesterol transporter Npc1 required for efficient autophagy^17^ (Figures 1D and 1E).

The rapid nature of cell death suggested an underlying metabolic collapse in autophagy-deficient cells.^18^ Loss of autophagy was previously shown to cause depletion of cellular metabolites although the mechanisms linking these metabolic defects to the cell death phenotype are poorly understood.^1^ To investigate the potential metabolic basis of cell death due to autophagy deficiency, we performed an unbiased metabolomics profiling of WT and *Atg5*^−/−^ MEFs after 16 h in galactose media, i.e., prior to the onset of cell death (Figures 1F–1H and S1L). In agreement with a previously proposed general defect in nucleic acid recycling in autophagy-deficient cells,^19^ a number of nucleotides were depleted in *Atg5*^−/−^ MEFs (Figures 1F–1H). By plotting the magnitude of change against the measure of significance, we identified the reduced form of nicotinamide (NAM) adenine dinucleotide (NADH) as the most significantly depleted metabolite in autophagy-deficient cells. NAD^+^, the oxidized form of the NAD, was also significantly decreased, suggesting that autophagy-deficient cells present with a depletion of the total pool of the dinucleotide (Figures 1F–1H). The NAD(H) deficit in *Atg5*^−/−^ MEFs was confirmed via a fluorescence-based assay (Figure 1I). Therefore, mitochondrial respiration in cells with autophagy deficiency is associated with the decline in NAD levels.

### Boosting intracellular NAD(H) rescues viability of autophagy-deficient cells

**Figure 2.**
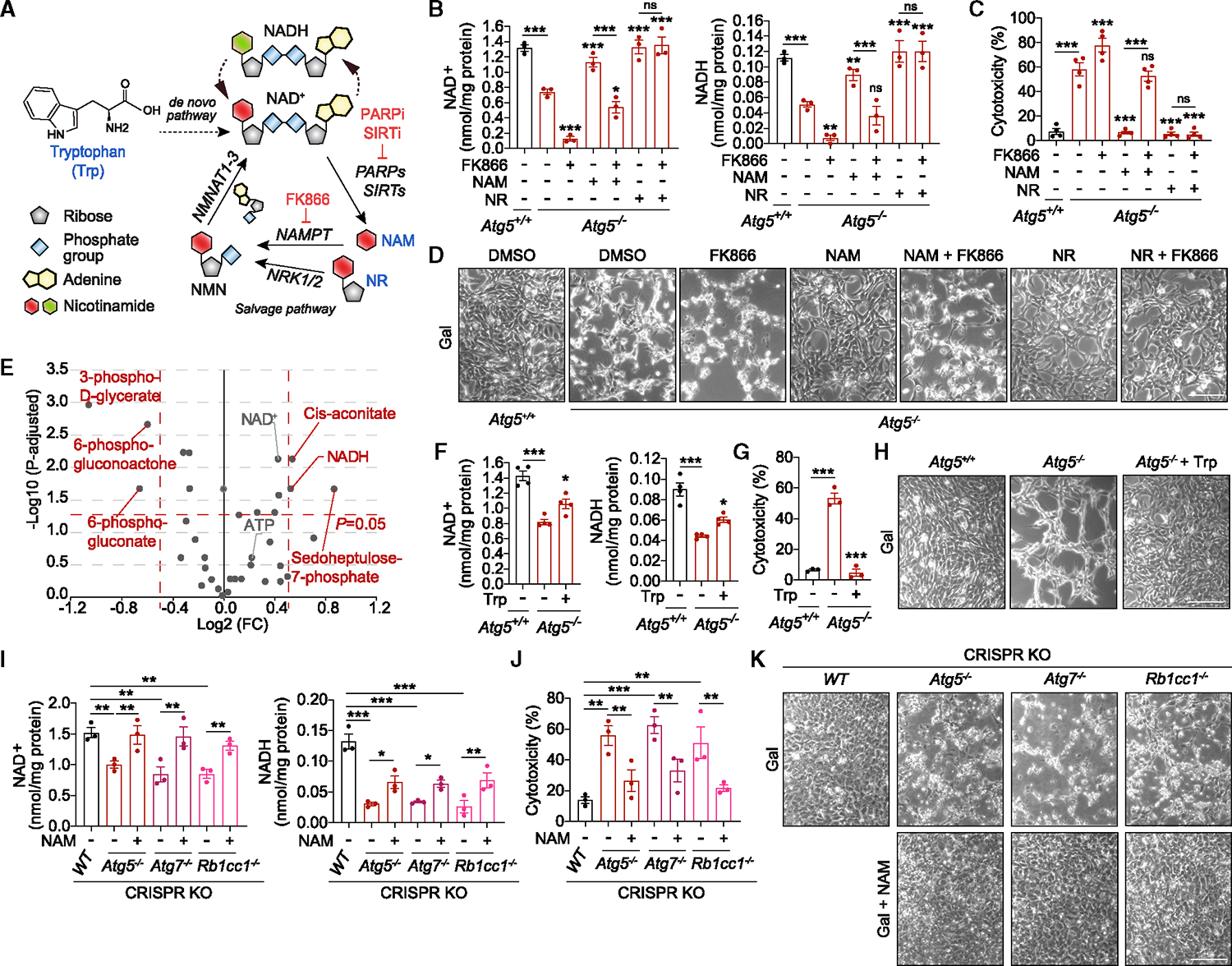
NAD(H) depletion contributes to cell death due to autophagy deficiency (A) Graphical representation of the NAD^+^ synthesis pathways. Enzyme inhibitors are highlighted in red. (B–D) Boosting NAD(H) levels via salvage pathway rescues cell death in respiring *Atg5*^−/−^ MEFs. Measurement of NAD^+^ and NADH (B), cytotoxicity assays (C), and phase contrast images (D) in *Atg5*^+/+^ and *Atg5*^−/−^ MEFs cultured for 20 h (B), 40 h (C), or 24 h (D) in gal medium supplemented with NAM or NR in the presence or absence of FK866. (E) Volcano plot representation of all analyzed metabolites in a pairwise comparison of *Atg5*^−/−^ + NAM to *Atg5*^−/−^ MEFs cultured in gal medium for 16 h. Thresholds are shown as dashed red lines. (F–H) Boosting NAD(H) levels via *de novo* pathway rescues cell death in respiring *Atg5*^−/−^ MEFs. Measurement of NAD^+^ and NADH (F), cytotoxicity assays (G), and phase contrast images (H) in *Atg5*^+/+^ and *Atg5*^−/−^ MEFs cultured for 20 h (F), 40 h (G), or 24 h (H) in gal medium supplemented with L-tryptophan (Trp). (I–K) NAD supplementation rescues cell death in various cell lines with autophagy deficiency. Measurement of NAD^+^ and NADH (I), cytotoxicity assays (J), and phase contrast images (K) in isogenic (CRISPR KO) non-targeted control (*WT*), *Atg5*^−/−^, *Atg7*^−/−^, and *Rbcc1*^−/−^ cell lines generated by the CRISPR-Cas9 system, cultured for 96 h (I), 144 h (J), or 110 h (K) in gal medium supplemented with NAM. Graphical data are mean ± SEM of n = 3–4 biological replicates as indicated (B, C, F, G, I, and J). p values were calculated by one-way ANOVA followed by multiple comparisons with the two-stage linear step-up procedure of Benjamini, Krieger, and Yekutieli (B, C, F, G, I, and J) or multiple t test with the original FDR method of Benjamini and Hochberg (E) on three independent experiments. *p < 0.05; **p < 0.01; ***p < 0.001; ns (non-significant) with respect to untreated *Atg5*^−/−^ MEFs or between the indicated groups. Scale bars, 200 μm in (D), (H), and (K). See also Figures S2 and S3.

We further investigated a potential role of NAD(H) in mediating cytotoxicity underlying autophagy deficiency. We first tested if the inhibition of NAD production affects cell viability. FK866, an inhibitor of NAM phosphoribosyltransferase (NAMPT) involved in NAD biosynthesis via a salvage pathway,^20^ compromised the viability of WT MEFs in galactose, but not glucose, medium, indicating that NAD(H) is a limiting factor for the survival of OXPHOS-dependent cells (Figures 2A, S2A, and S2B). To test whether boosting intracellular NAD(H) levels is sufficient to rescue the viability of autophagy-deficient cells, we utilized the native cellular capacity for NAD synthesis (Figure 2A). Supplementation of bioavailable NAD precursors, NAM or NAM riboside (NR), led to the recovery of intracellular NAD^+^ and NADH levels and rescued viability of *Atg5*^−/−^ MEFs (Figures 2B–2D). The effect of NAM (but not NR) on cell survival was abrogated by FK866, consistent with the dependency on NAMPT for the conversion of this precursor to NAD(H) via the salvage pathway (Figures 2A–2D). An unbiased metabolomics profiling of *Atg5*^−/−^ MEF in galactose medium, treated with or without NAM, was performed to assess any correlation between the rescue of cell death and recovery of intracellular metabolites. By plotting the magnitude of rescue against the measure of significance, we found NADH to be the only metabolite that correlated with *Atg5*^−/−^ MEF viability, i.e., it was first found to be significantly depleted in *Atg5*^−/−^ MEF (Figures 1F and 1G) and then significantly restored by NAM supplementation (Figures 2E, S2C, and S2D), with NAD^+^ following a similar trend. Therefore, the rescue of cell death is mediated by increased NAD(H), but not, for example, ATP (Figures 2E, S2C, and S2D). The role of NAD(H) was further supported by NAD supplementation via *de novo* pathway using L-tryptophan (Figure 2A), which partially recovered NAD(H) levels and which was sufficient to rescue cell death (Figures 2F–2H). Importantly, NAD(H) depletion as a driver of cell death was confirmed in several genome-edited cell models of autophagy deficiency (Figures 2I–2K). Taken together, the data show that the NAD salvage and *de novo* pathways remain active in autophagy-deficient cells, and therefore, NAD(H) depletion is likely to be mediated by its increased consumption.

### Hyperactivation of NADases drives NAD(H) depletion in autophagy-deficient cells

**Figure 3.**
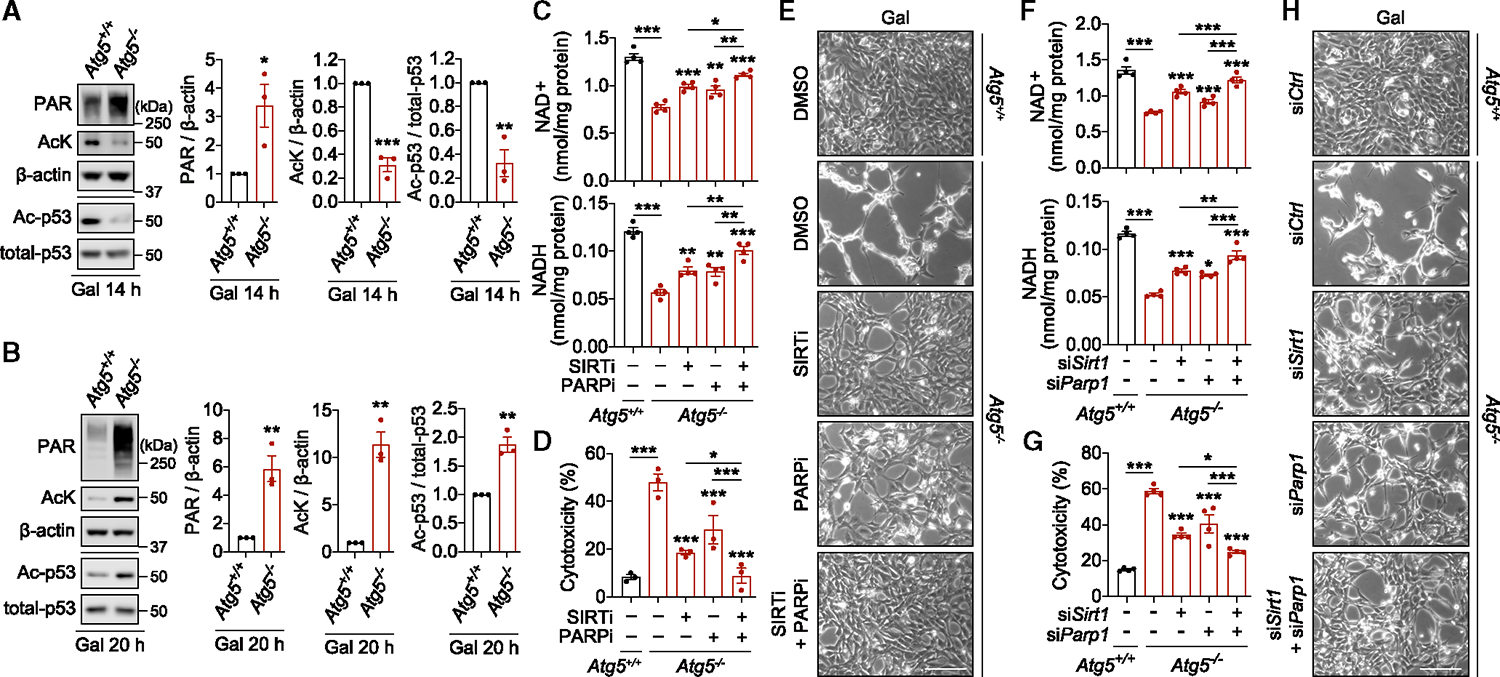
NAD(H) depletion due to the hyperactivation of NADases in autophagy-deficient cells (A and B) Kinetic hyperactivation of NADases in respiring *Atg5*^−/−^ MEFs. Immunoblot analyses for poly(ADP-ribose) (PAR), acetylated lysine (AcK), acetylated p53 (Ac-p53), and total p53 as indicators of PARP and SIRT activities in *Atg5*^+/+^ and *Atg5*^−/−^ MEFs cultured in gal medium for 14 h (A) or 20 h (B). (C–H) Chemical or genetic inhibition of NADases activity rescues NAD(H) levels and cell death in respiring *Atg5*^−/−^ MEFs. Measurement of NAD^+^ and NADH levels (C and F), cytotoxicity assays (D and G), and phase contrast images (E and H) in *Atg5*^+/+^ and *Atg5*^−/−^ MEFs treated with sirtinol (SIRTi), olaparib, (PARPi) or solvent (DMSO); and in *Atg5*^+/+^ and *Atg5*^−/−^ MEFs transfected with *Control, Sirt1*, or *Parp1* siRNA, after 20 h (C and F), 40 h (D and G), or 24 h (E and H) culture in gal medium. Graphical data are mean ± SEM of n = 3–4 biological replicates as indicated (A–D, F, and G). p values were calculated by unpaired two-tailed Student’s t test (A and B) or one-way ANOVA followed by multiple comparisons with the two-stage linear step-up procedure of Benjamini, Krieger, and Yekutieli (C, D, F, and G) on three independent experiments. *p < 0.05; **p < 0.01; ***p < 0.001 with respect to untreated *Atg5*^−/−^ MEFs or between the indicated groups. Scale bars, 200 μm in (E) and (H). See also Figure S3.

We next investigated the mechanism of NAD(H) depletion in autophagy-deficient cells. Activities of two main classes of NAD-consuming enzymes, poly-ADP-ribose polymerases (PARPs) and deacetylases of sirtuin family (SIRTs),^21^ were increased in *Atg5*^−/−^ MEFs after 14 h culture in galactose medium. This was evident from the elevated levels of poly-ADP-ribosylation (PARylation) and reduced protein acetylation including acetylated-p53, respectively (Figure 3A). PARylation, but not deacetylation, activity remained elevated after 20 h of culture (Figure 3B), consistent with the reliance of SIRTs but not PARPs on high cellular NAD^+^ levels.^9^

We did not find evidence for PARP hyperactivation as a direct cause of cell death, which is mediated by the mitochondria-to-nucleus translocation of apoptosis-inducing factor (AIF) (Figure S3A). Therefore, we hypothesized that the loss of cell viability is triggered by NAD(H) exhaustion due to uncontrolled NAD^+^ cleavage. Indeed, pharmacological inhibition of SIRTs with sirtinol or PARPs with olaparib partially rescued both intracellular NAD(H) levels and cell viability of *Atg5*^−/−^ MEFs (Figures 3C–3E and S3B). The role of NADases was further validated by siRNA-mediated knockdown of *Sirt1* or *Parp1*, indicating involvement of these enzymes in NAD(H) depletion and cell death (Figures 3F–3H and S3C). Simultaneous suppression of SIRTs and PARPs rescued NAD(H) levels and cell viability over and above the effect achieved with the inhibition of either one of the enzymes, supporting our conclusion that these two classes of NADases are jointly contributing to NAD(H) depletion (Figures 3C–3H, S3B, and S3C).

NAM and NR also suppressed PARP and/or SIRT activities consistent with their known capacity as competitive inhibitors^21^ (Figure S3D). However, several lines of evidence indicate that the inhibition of NADases is not the mechanism by which NAD precursors rescue cell viability. For example, FK866 suppressed PARylation/deacetylation, presumably by reducing NAD(H) levels, but cell death was instead further enhanced (Figures 2B–2D and S3D). Additionally, NAM was unable to rescue NAD(H) levels and cell viability in the presence of the NAMPT inhibitor, while PARylation/deacetylation activity was strongly suppressed (Figures 2B–2D and S3D). Finally, boosting NAD levels via the *de novo* pathway by L-tryptophan supplementation had no inhibitory effect on PARPs/SIRTs activities (Figures 2F–2H and S3E). We therefore conclude that while hyperactivation of PARPs/SIRTs drives NAD(H) depletion, boosting NAD levels acts downstream of NADase activities to rescue cell death.

### Loss of mitochondrial quality control triggers the cascade leading to NAD(H) depletion

**Figure 4.**
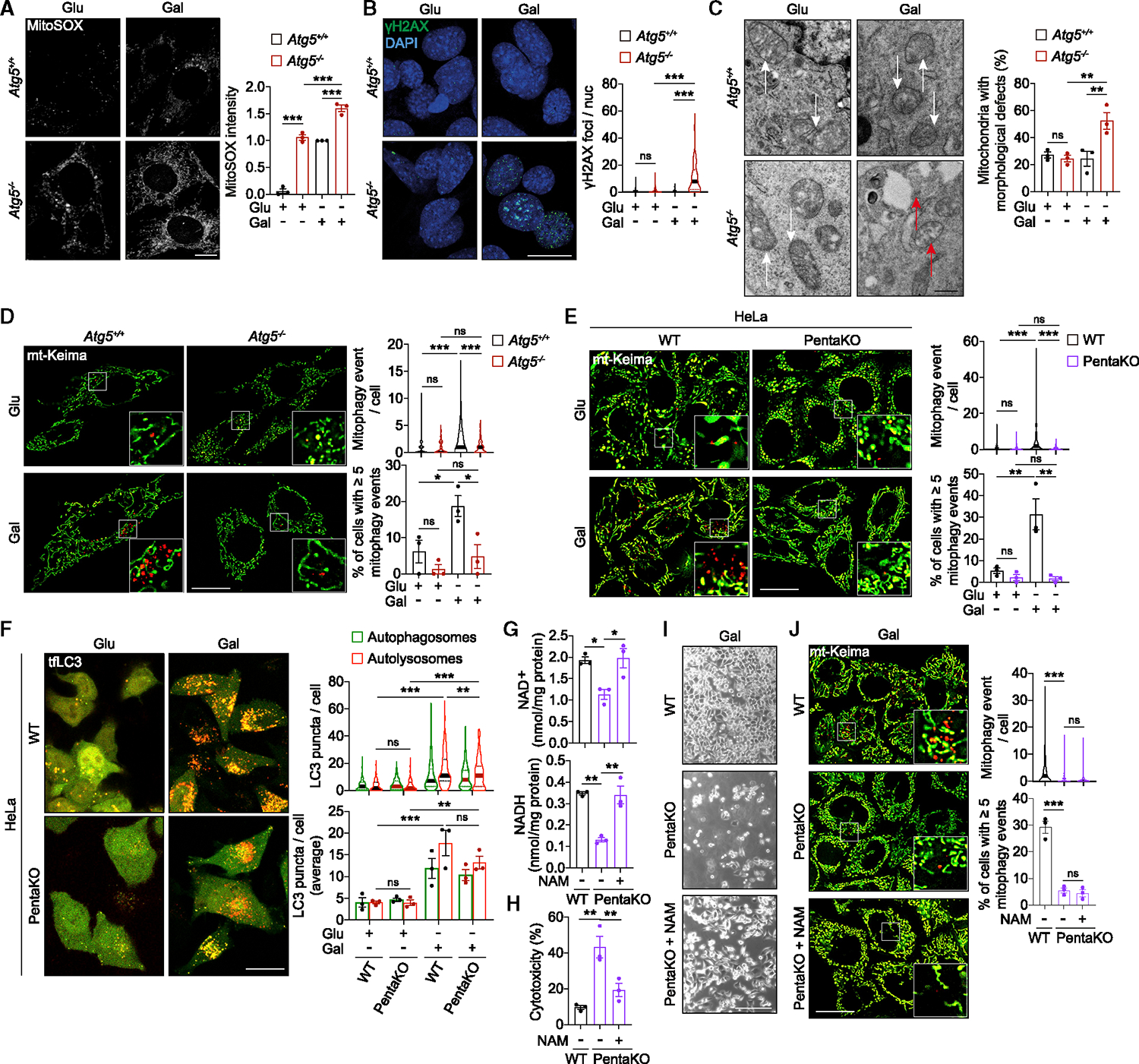
Loss of mitochondrial quality control contributes to the depletion of NAD(H) and cell death (A and B) Mitochondrial ROS and DNA damage are induced in respiring *Atg5*^−/−^ MEFs. Fluorescence microscopy images and quantification of MitoSOX staining (A) or γH2AX immunostaining (B) of *Atg5*^+/+^ or *Atg5*^−/−^ MEFs cultured in glu or gal medium for 24 h. (C) Transmission electron micrographs of *Atg5*^+/+^ or *Atg5*^−/−^ MEFs cultured in glu or gal medium for 24 h. White arrows: healthy mitochondria; red arrows: mitochondria with aberrant morphology. (D and E) Mitophagy defect in *Atg5*^−/−^ MEFs and PentaKO HeLa cells. Fluorescence microscopy images and quantification of mitophagy of *Atg5*^+/+^ or *Atg5*^−/−^ MEFs (D) and WT or PentaKO HeLa cells (E), expressing mt-mKeima and cultured in glu or gal medium for 24 h (D) or 96 h (E). (F) Autophagy is activated in respiring PentaKO cells. Confocal fluorescence microscopy images and quantification of autophagosomes and autolysosomes in WT or PentaKO HeLa cells expressing mRFP-GFP tandem fluorescent-tagged LC3 (tfLC3) cultured in glu or gal medium for 96 h. (G–I) Loss of mitophagy contributes to NAD(H) depletion and cell death in respiring PentaKO HeLa cells. Measurement of NAD^+^ and NADH (G), cytotoxicity assays (H), and phase contrast images (I). (G) in WT or Penta KO HeLa cells cultured for 144 h (H), 120 h (I), or 110 h (G) in gal medium supplemented with 1 mM NAM. (J) Mitophagy defect is upstream of NAD(H) depletion. Fluorescence microscopy images and quantification of mitophagy in WT or PentaKO HeLa cells expressing mt-mKeima and cultured for 96 h in gal medium supplemented with 1 mM NAM. Data are mean ± SEM (A, C–H, and J) or displayed as cell popular violin plots (B, D–F, and J). p values were calculated by one-way ANOVA followed by multiple comparisons with the two-stage linear step-up procedure of Benjamini, Krieger, and Yekutieli on three independent experiments (A–H and J). *p < 0.05; **p < 0.01; ***p < 0.001; ns (non-significant). Scale bars: 10 μm in (A); 20 μm in (B), (D)–(F), and (J); 500 nm in (C); and 200 μm in (I). See also Figure S4.

SIRTs and PARPs are activated by reactive oxygen species (ROS) and DNA damage^9^ that were found to be significantly elevated in respiring *Atg5*^−/−^ MEFs (Figures 4A, 4B, and S4A), likely resulting from mitochondrial dysfunction. Indeed, OXPHOS-dependent *Atg5*^−/−^ MEFs displayed disrupted mitochondrial morphology, altered levels of electron transport chain supercomplexes, and impaired generation of ATP via OXPHOS (and increased reliance on glycolysis in glucose media) (Figures 4C, S4B, and S4C). We rationalized that the accumulation of damaged mitochondria resulted from the loss of selective autophagic clearance of dysfunctional mitochondria (mitophagy) (Figures 4D and S4D).^8,22^ To test this, we used a cell line previously shown to be defective in damage-induced mitophagy due to knockout of 5 selective autophagy receptors (SARs) (HeLa PentaKO).^23^ Enforcing mitochondrial respiration by galactose medium strongly increased basal mitophagy and bulk autophagy in control HeLa cells (Figures 4E and 4F). Instead, PentaKO maintained autophagy flux but lost the mitophagy capacity, which was sufficient to recapitulate the cell death phenotype in galactose medium (Figures 4E–4I). Supplementation with NAM rescued NAD(H) levels and cell death without rescuing mitophagy in PentaKO cells (Figures 4G–4J).

**Figure 5.**
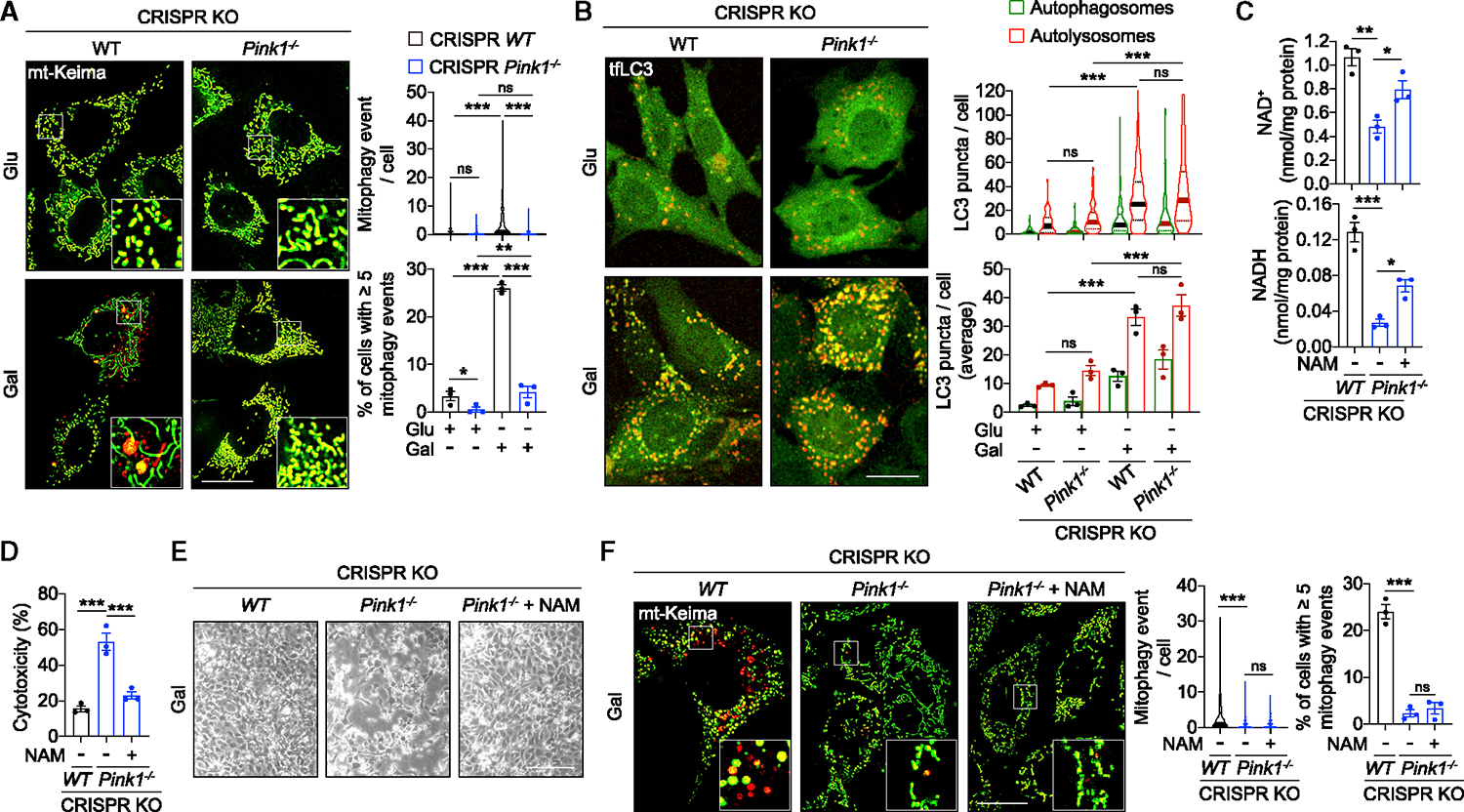
Loss of PINK1 supresses mitophagy activated by galactose media culture and recapitulates NAD(H) depletion and cell death (A) Fluorescence microscopy images and quantification of mitophagy of isogenic non-targeted control (CRISPR *WT*) or *Pink1*^−/−^ (CRISPR *Pink1*^−/−^) MEFs generated by the CRISPR-Cas9 system, expressing mt-mKeima and YFP-Parkin, cultured in glu or gal medium for 96 h. (B) Confocal fluorescence microscopy images and quantification of autophagosomes and autolysosomes in CRISPR *WT* or *Pink1*^−/−^ MEFs expressing tfLC3 cultured in glu or gal medium for 96 h. (C–E) Measurement of NAD^+^ and NADH (C), cytotoxicity assays (D), and phase contrast images (E) of CRISPR *WT* or *Pink1*^−/−^ MEFs cultured for 134 h (C), 144 h (D), or 168 h (E) in gal medium supplemented with 5 mM NAM. (F) Fluorescence microscopy images and quantification of mitophagy in CRISPR *WT* or *Pink1*^−/−^ MEFs expressing mt-mKeima and YFP-Parkin cultured for 96 h in gal medium supplemented with 5 mM NAM. Data are mean ± SEM (A–D and F) or displayed as cell popular violin plots (A, B, and F). p values were calculated by one-way ANOVA followed by multiple comparisons with the two-stage linear step-up procedure of Benjamini, Krieger, and Yekutieli on three independent experiments (A–D and F). *p < 0.05; **p < 0.01; ***p < 0.001; ns (non-significant). Scale bars: 20 μm in (A), (B), and (F) and 200 μm in (E). See also Figure S5.

**Figure 6.**
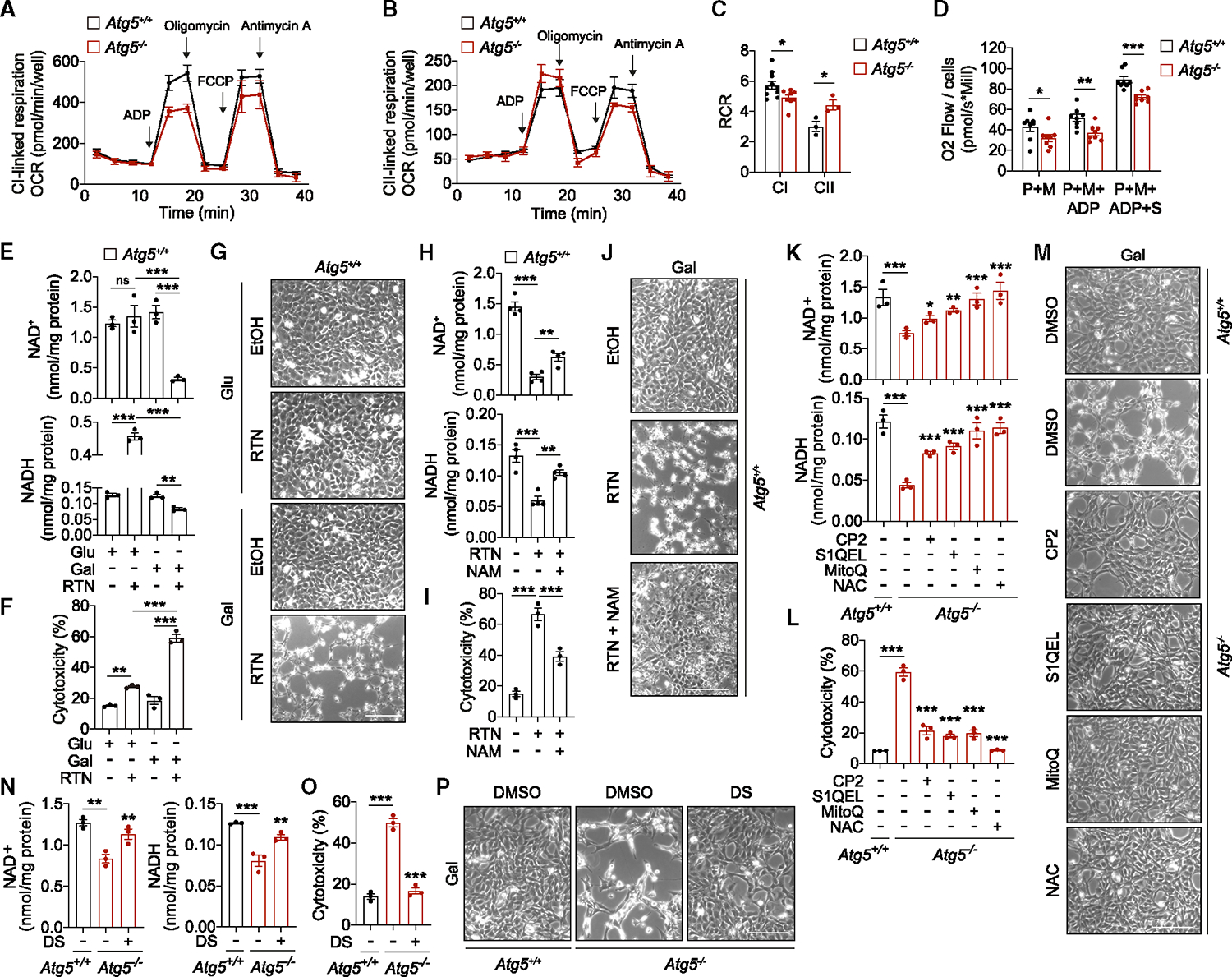
Respiration via dysfunctional mitochondrial CI is upstream of NAD(H) depletion and loss of viability in autophagy-deficient cells (A–D) Mitochondrial CI is dysfunctional in *Atg5*^−/−^ MEFs. (A–C) CI- and CII-linked respiration were assessed by measuring oxygen consumption rate (OCR) in permeabilized *Atg5*^+/+^ and *Atg5*^−/−^ MEFs in assay buffer supplemented with either CI substrates, pyruvate, and malate (P+M) (A) or CII substrate and succinate (S) (B). Oligomycin, FCCP, and animycin A were added at the indicated times during OCR measurements (A and B). Respiratory control ratios (RCRs) indicate a capacity for substrate oxidation and ATP turnover (C). (D) Respirometry analysis of *Atg5*^+/+^ and *Atg5*^−/−^ MEFs cultured in gal medium for 20 h was performed by sequential additions of CI substrates P+M, ADP, and S into suspension of 1 million permeabilized cells with digitonin. (E–J) Rotenone causes NAD(H) depletion and cell death in respiring wild-type cells. Measurement of NAD^+^ and NADH (E and H), cytotoxicity assays (F and I), and phase contrast images (G and J) in *Atg5*^+/+^ MEFs cultured for 6 h (E and H), 12 h (F and I), and 9 h (G and J) in glu or gal medium supplemented with 1 μM rotenone (RTN) in the presence or absence of 5 mM NAM. Cells were pre-treated with NAM for 16 h prior to RTN treatment (H–J). (K–P) Chemical interventions in CI dysfunction, mitochondrial ROS, and boosting CII activity rescue NAD(H) and cell death phenotypes. Measurement of NAD^+^ and NADH (K and N), cytotoxicity assays (L and O), and phase contrast images (M and P) in *Atg5*^+/+^ and *Atg5*^−/−^ MEFs cultured for 20 h (K and N), 40 h (L and O), or 24 h (M and P) in gal medium supplemented with CP2, S1QEL2.2 (S1QEL), MitoQ, or N-acetyl-L-cysteine (NAC) (K–M) or with dimethyl succinate (DS) (N–P). Graphical data are mean ± SEM of biological replicates (n = 3) (A and B) or more replicates as indicated (C–F, H, I, K, L, N, and O). p values were calculated by multiple t test (C and D) or one-way ANOVA followed by multiple comparisons with the two-stage linear step-up procedure of Benjamini, Krieger, and Yekutieli (E, F, H, I, K, L, N, and O) on three independent experiments. *p < 0.05; **p < 0.01; ***p < 0.001; ns (non-significant) with respect to untreated *Atg5*^−/−^ MEFs or between the indicated groups. Scale bars, 200 μm in (G), (J), (M), and (P). See also Figure S6.

As an alternative model of mitophagy deficiency, we generated MEFs with CRISPR-Cas9 knockout of *Pink1*. Recruitment of the ubiquitin ligase Parkin to damaged mitochondria (e.g., caused by antimycin/oligomycin) by PINK1 is essential for the stress-induced mitophagy (Figures S5A and S5B).^23^ Loss of PINK1 also suppressed Parkin recruitment and mitophagy in unstressed cells both in glucose and galactose medium (Figures 5A and S5C). Like PentaKO cells, loss of mitophagy in *Pink1*^−/−^ MEFs did not affect general autophagy flux (Figures 4F and 5B). At the same time, in galactose medium, loss of PINK1 was sufficient to trigger NAD(H) depletion and cell death, which were significantly rescued by NAM supplementation without rescuing mitophagy (Figures 5C–5F and S5D). Together with the data obtained in PentaKO cells, these results place the loss of mitochondrial quality control upstream of the metabolic and viability deficits in autophagy deficiency. Damaged mitochondrial CI is the main driver of mitochondrial ROS generation,^24^ which we speculated to trigger cell stress in autophagy-deficient cells. Indeed, measurements of mitochondrial respiration in permeabilized cells indicated CI-specific deficiency in *Atg5*^−/−^ MEFs that was partially compensated by increased complex II (CII)-dependent activity (Figures 6A–6D). To test whether CI damage is sufficient to recapitulate the phenotypes of autophagy-deficient cells, we treated WT MEFs with rotenone (RTN) in glucose and galactose media. Interestingly, while in glucose medium, RTN treatment increased the NADH levels consistent with its function as an inhibitor of the CI NADH dehydrogenase activity,^24^ in galactose medium, RTN caused depletion of both NAD and NADH (Figure 6E). RTN also recapitulated the cell death phenotype of autophagy-deficient cells in galactose medium (Figures 6F and 6G). The relevance of NAD(H) depletion to cell death in this model was indicated by NAM supplementation, which significantly rescued both NAD(H) depletion and cytotoxicity induced by RTN (Figures 6H–6J).

Next, several pharmacological interventions were used to test the role of CI deficiency in autophagy-deficient cells, including CP2, a mild inhibitor of CI (without ROS induction associated with RTN);^25,26^ S1QEL2.2, a specific inhibitor of ROS production by CI;^27^ MitoQ, a mitochondria-targeted ROS scavenger;^28^ and N-acetyl-L-cysteine (NAC), a general antioxidant.^29^ All the treatments rescued DNA damage, PARP and Sirtuin hyperactivation, NAD(H) depletion, and cell death of autophagy-deficient cells in galactose medium (Figures 6K–6M, S6A, and S6B). An alternative strategy to reduce metabolic reliance on CI is boosting CII-dependent respiration, e.g., using succinate as a substrate.^30^ Supplementation of dimethyl succinate (DS) in galactose medium was sufficient to prevent NAD(H) depletion by NADases and cell death (Figures 6N–6P and S6C). Taken together, dysfunctional mitochondrial CI in respiring autophagy-deficient cells drives cellular stress by producing elevated levels of ROS, which via hyperactivation of NADases causes NAD(H) depletion and cell death.

### NAD(H) depletion causes cell death via mitochondrial depolarization

**Figure 7.**
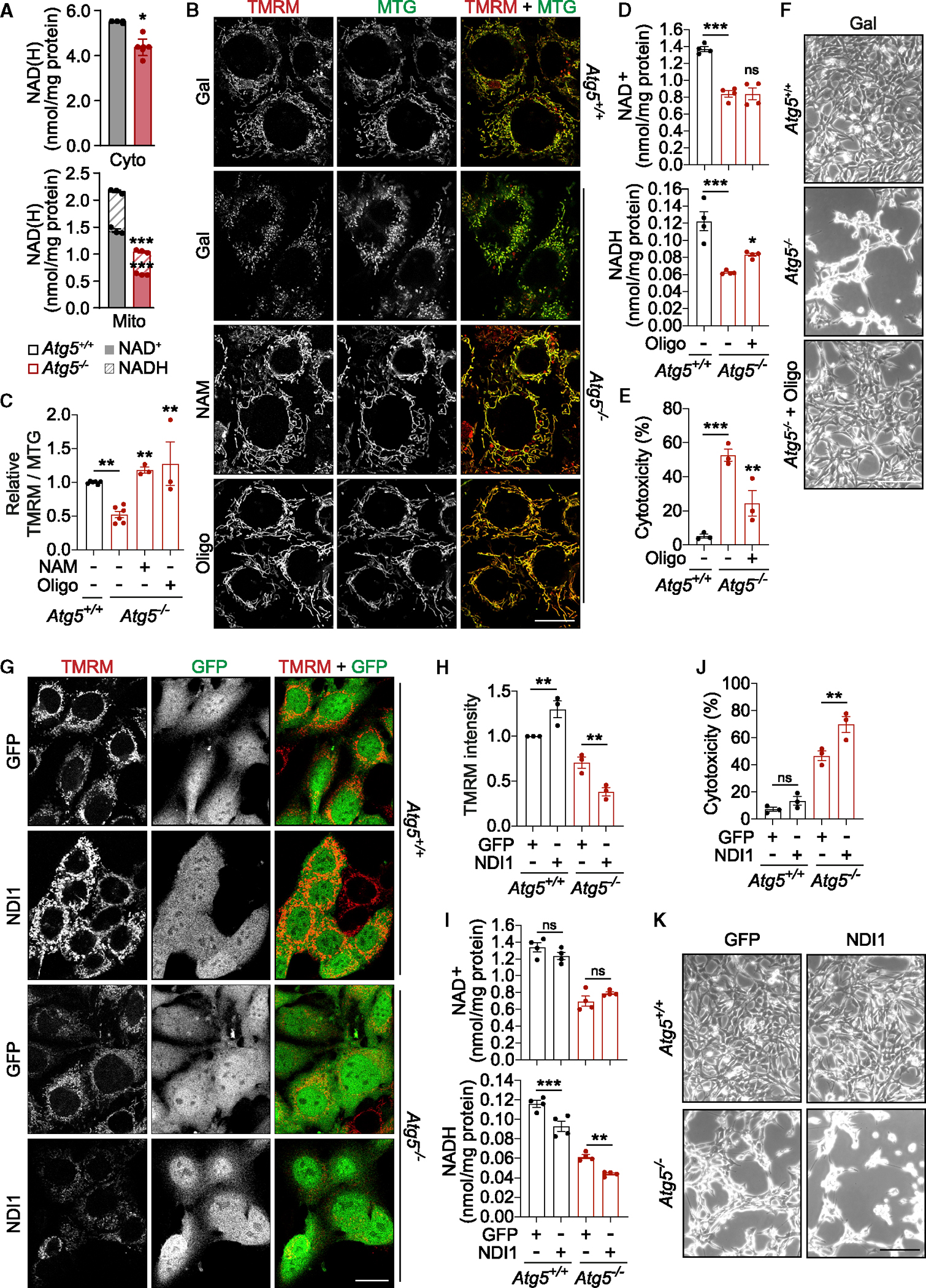
Depletion of mitochondrial NADH and membrane potential mediates cell death due to autophagy deficiency (A) Mitochondrial NADH is depleted in respiring *Atg5*^−/−^ MEFs. Measurement of NAD^+^ and NADH levels in cytoplasmic (cyto) and mitochondrial (mito) fractions of *Atg5*^+/+^ and *Atg5*^−/−^ MEFs after 20 h culture in gal medium. (B and C) Mitochondrial depolarization in respiring *Atg5*^−/−^ MEFs is rescued by NAD supplementation. Confocal fluorescence images of live cells after 20 h culture in gal medium supplemented with NAM or oligomycin (Oligo) and co-stained with TMRM and MTG (B). ΔΨm quantified as a ratio of TMRM to MTG (C). (D–F) Mitochondrial hyperpolarization by oligomycin rescues cell death with NADH but not NAD^+^ restoration in respiring *Atg5*^−/−^ MEFs. Measurement of NAD^+^ and NADH levels (D), cytotoxicity assays (E), and phase contrast images (F) in *Atg5*^+/+^ and *Atg5*^−/−^ MEFs treated with Oligo or solvent (DMSO) after 20 h (D), 40 h (E), or 24 h (F) culture in gal medium. (G–K) Boosting NADH consumption by alternative NADH dehydrogenase NDI1 augments mitochondrial depolarization and cell death phenotypes in respiring *Atg5*^−/−^ MEFs. Confocal fluorescence images of live cells stained with TMRM (G), ΔΨm quantified as the relative intensity of TMRM in GFP positive cells (H), measurement of NAD^+^ and NADH levels (I), cytotoxicity assays (J), and phase contrast images (K) in *Atg5*^+/+^ and *Atg5*^−/−^ MEFs stably expressing NDI1-IRES-GFP (NDI1) or GFP after 20 h (G–I), 40 h (J), or 24 h (K) culture in gal medium. Graphical data are mean ± SEM of n = 3–4 biological replicates as indicated (A, C–E, and H–J). p values were calculated by unpaired two-tailed Student’s t test (A) or one-way ANOVA followed by multiple comparisons with the two-stage linear step-up procedure of Benjamini, Krieger, and Yekutieli (C–E and H–J) on three independent experiments. *p < 0.05; **p < 0.01; ***p < 0.001; ns (non-significant) with respect to untreated *Atg5*^−/−^ MEFs or between the indicated groups. Scale bars: 20 μm in (B) and (G) and 200 μm in (F) and (K). See also Figures S6 and S7.

We found that dysfunctional mitochondrial CI and mitochondrial respiration could trigger cell death in autophagy-deficient cells (Figures 6 and S1C–S1H). NADH was also identified as the most significantly depleted metabolite in autophagy-deficient cells and the only metabolite that correlated with *Atg5*^−/−^ MEF viability (Figures 1F, 1G, 2E, S2C, and S2D). Given that NADH is essential for mitochondrial respiration by providing electrons for mitochondrial CI,^30^ we next investigated the mechanism linking NADH depletion and cell death. NADH was predominantly detected in a mitochondria-enriched cell fraction where it was depleted in *Atg5*^−/−^ MEFs cultured in galactose medium (Figures 7A and S6D). Oxidation of NADH generates mitochondrial membrane potential (ΔΨm) across the inner mitochondrial membrane, and we hypothesized that the depletion of mitochondrial NADH can trigger mitochondrial depolarization and apoptosis.^8,31^ Indeed, boosting NAD(H) levels with NAM rescued membrane depolarization detected in respiring *Atg5*^−/−^ MEFs (Figures 7B and 7C). Furthermore, preventing dissipation of ΔΨm by suppressing ATP synthase activity (using a low dose of oligomycin),^32^ partially prevented the depletion of NADH and rescued cell death (Figures 7B–7F). Consistent with no effect of oligomycin on NAD^+^ levels (Figure 7D), SIRT and PARP activities remained unaffected (Figure S6E), indicating that oligomycin acted downstream of NADases. To further test the role of mitochondrial NADH in the cell death phenotype, we overexpressed a non-proton-pumping alternative NADH oxidase, NDI1.^33^ In respiring autophagy-deficient cells, the overexpression of NDI1 led to an increased oxidation state of the NAD(H) pool, which enhanced the dissipation of ΔΨm and the apoptotic phenotype (Figures 7G–7K and S6F). We conclude that NADH is the limiting factor in the survival of autophagy-deficient cells.

Since *Atg5*^−/−^ MEFs manifested with general nucleotide depletion (Figures 1F–1H), we supplemented cells in galactose medium with five nucleosides and found that all were able to restore cell viability, while NAD(H) levels were not rescued (Figures S7A–S7C). Therefore, NAD(H) decline is independent of the previously proposed purine/pyrimidine depletion mechanism of cell death in autophagy-deficient tumor-derived cells.^19^ However, nucleoside supplementation prevented the dissipation of ΔΨm in respiring *Atg5*^−/−^ MEFs, which would explain the rescue of cell death (Figures S7D and S7E). Therefore, the ability of nucleosides to preserve membrane potential, for example, by previously reported inhibition of mitochondrial uncoupling proteins,^34^ is an additional mechanism acting downstream of NAD(H) depletion and preventing cell death due to autophagy deficiency.

### Evolutionarily conserved role of autophagy in maintaining NAD(H) levels

**Figure 8.**
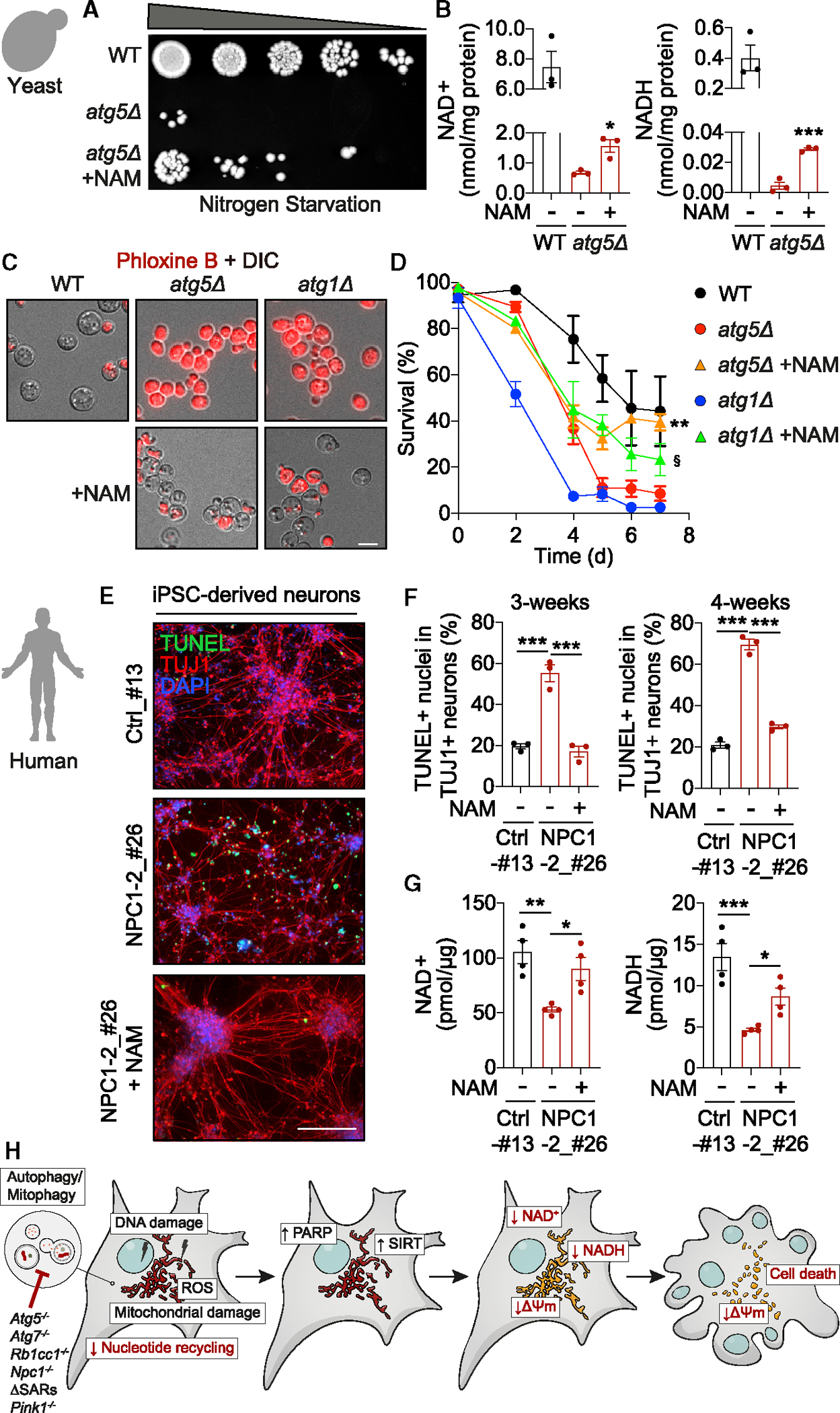
Evolutionarily conserved role of autophagy in the maintenance of NAD(H) (A and B) Spot-testing (5-fold serial dilutions) of S288C WT and *atg5Δ* yeast strains on nutrient-rich agar media (A) and measurement of NAD^+^ and NADH levels (B) following 5 days (A) or 3 days (B) of nitrogen starvation in the presence of 10 mM NAM or solvent (H_2_O). Image is a representative of n = 4 experiments. (C and D) Representative images (overlay of DIC and fluorescence images) (C) and quantification of cell viability (D) of BY4741 (WT), ScPPS2 (*atg5Δ*), and sSUN99 (*atg1Δ*) yeast strains cultured in SD-N medium supplemented with 10 mM NAM for 6 days (C) or the indicated times (D) followed by Phloxine B staining. (E–G) Fluorescence images (E), quantification of TUNEL^+^ apoptotic nuclei in TUJ1^+^ cells (F), and measurement of NAD^+^ and NADH levels (G) in control (Ctrl_#13) and NPC1 (NPC1–2_#26) iPSC-derived neurons after 3 (F and G) or 4 (F) weeks of neuronal differentiation, where NPC1 neurons were treated with or without NAM for the last 6 days. (H) Schematic representation of the mechanism of NAD(H) depletion leading to mitochondrial depolarization and apoptosis in respiring autophagy-deficient cells. SARs, selective autophagy receptors. Graphical data are mean ± SEM of n = 3 (B, D, and F) or 4 (G) biological replicates. p values were calculated by unpaired two-tailed Student’s t test (B and D within dataset on day 6) or one-way ANOVA followed by multiple comparisons with the two-stage linear step-up procedure of Benjamini, Krieger, and Yekutieli (F and G) on three or four independent experiments. *p < 0.05; **p < 0.01; ***p < 0.001; ns (non-significant) with respect to untreated *atg5Δ* yeast or between the indicated groups. ^§^p < 0.05 with respect to *atg1Δ* yeast. Scale bars: 5 μm in (C) and 100 μm in (E). See also Figure S8.

Autophagy is required for the survival of eukaryotic organisms from yeast to man.^7^ We investigated whether the role of autophagy in the maintenance of intracellular NAD pools is evolutionarily conserved and it underlies the importance of autophagy for cell survival. Nitrogen-deprived *Saccharomyces cerevisiae* yeast are dependent on mitochondrial respiration, and autophagy deficiency causes accumulation of ROS, loss of respiratory capacity, and cell death.^3^ We found that reduced survival of nitrogen-deprived *atg5Δ* yeast was associated with a striking depletion of NAD(H) levels (Figures 8A and 8B). Supplementation with NAM partially rescued NAD(H) levels and significantly improved cell survival (Figures 8A and 8B, note 5-fold culture dilutions used for spotting). Phloxine B staining validated the effect of NAM on cell viability of *atg5Δ* as well as another autophagy-deficient *atg1Δ* yeast strain (Figures 8C and 8D). Importantly, NAM was not providing a nitrogen source, as it did not rescue growth in starvation conditions (Figure S8A), and instead, conversion of NAM to NAD^+^ was required as knockout of *pnc1* mediating this process in yeast blocked the rescue of *atg5Δ* cell viability (Figures S8B and S8C). NAM did not rescue the defect in autophagic processing of Atg8 (homolog of human LC3) in *atg5Δ* and *atg1Δ* yeast, suggesting that NAM-mediated protection from loss of viability did not occur due to the reconstitution of autophagy (Figures S8D–S8F). These findings are consistent with the protective effects of NAD-boosting strategies downstream of autophagy dysfunction as observed in our mouse cell-based models.

Finally, we investigated disease-affected human neurons differentiated from patient-derived human induced pluripotent stem cells (hiPSCs) of a neurodegenerative lysosomal storage disorder, Niemann-Pick type C1 (NPC1) disease. As previously reported,^35^ NPC1 neurons presented with a severe autophagic defect (Figures S8G–S8I) and cell death between 3 and 4 weeks after differentiation (Figures 8E and 8F). Similar to our findings in other autophagy-deficient models, autophagy dysfunction in NPC1 neurons correlated with NAD(H) depletion (Figure 8G). Treatment with NAM was able to rescue both NAD(H) levels and cell death phenotype in NPC1 neurons (Figures 8E–8G). Taken together, our findings provide a blueprint for mechanistic and translational studies of NAD manipulation as a therapeutic strategy in a wide range of human diseases with autophagy dysfunction.

## DISCUSSION

We found that autophagy deficiency in actively respiring cells is sufficient to cause cell death. While damaged mitochondria, and particularly CI, trigger cellular stress, the cell death is indirectly mediated by the hyperactivity of stress-responsive NAD-dependent enzymes of PARP and SIRT families, which ultimately leads to the loss of NAD(H). Exhaustion of NADH within mitochondria appears to be the weakest link in this chain of events, as it triggers mitochondrial membrane depolarization and activation of apoptosis (Figure 8H). This model, where the demise of autophagy-deficient cells is mediated by the persistent activation of stress response pathways, is conceptually analogous to the previously proposed mechanism involving the hyperactivation of Nrf2.^36^ How this and other mechanisms, such as depletion of the general nucleotide pool in autophagy-deficient cells,^19^ integrate with the critical role of NAD depletion during autophagy deficiency in different pathologies remains to be investigated (Figure 8H).

Ageing and age-related diseases have long been associated with decline in both autophagy and NAD levels.^37^ Nutritional or pharmacological activation of autophagy is currently a subject of intense research for the development of small molecule modulators.^38^ However, due to the varied nature of autophagy dysfunction in genetic and age-related sporadic diseases, including the impairment in lysosomal degradative capability, the development of a universal modulator remains unlikely.^38–40^ In contrast, boosting the levels of NAD by precursor supplementation in animal models was found to have a positive impact on age-related pathologies, which is at least in part mediated by the upregulation of autophagy/mitophagy.^37^ Crucially, our data show that autophagy is, in turn, required for NAD maintenance and that increasing NAD levels protects cells by preventing the loss of ΔΨm even in the absence of functional autophagy. Our conclusions are consistent with the previous findings indicating that boosting NAD may protect against the pathology associated with the impairment of lysosomal function.^41^

Another important conclusion from our study is that the hyperactivation of PARPs and SIRTs, which in healthy state, maintain cellular homeostasis and promote organismal longevity, may become a culprit in the demise of cells and organisms when the autophagy-lysosome pathway is impaired. Therefore, caution needs to be exercised when considering interventions aiming to boost the activity of these pathways in pathological situations associated with autophagy-lysosome dysfunction. The indirect mechanism leading to the viability defect is likely to be applicable to a range of pathological states associated with the dysfunction of autophagy, mitophagy, lysosomal impairment, or mitochondrial damage observed in human ageing and age-related diseases. As such, our investigations define a mechanism linking autophagy-lysosome pathway, NAD metabolism, and ageing. Finally, our studies point toward several therapeutic or lifestyle interventions (e.g., nutritional regimens reducing reliance on mitochondrial CI) to alleviate cellular pathology in a range of diseases associated with autophagic, lysosomal, and mitochondrial dysfunction.

### Limitations of the study

In this study, we demonstrated an evolutionarily conserved role of autophagy/mitophagy in the maintenance of NAD levels and cell survival. The experiments were carried out using immortalized mouse fibroblast models carrying genetic deletions/mutations of various autophagy/mitophagy genes. In addition to PARP and SIRT enzymes that are shown in our study to be responsible for the uncontrolled depletion of NAD pools in autophagy-deficient fibroblasts, other NADases are expressed in different cells and tissues and need to be investigated for their interplay with autophagy in the future. Confirmation of the role of autophagy in the maintenance of NAD levels and cell survival was done in yeast as well as human neurons. However, we did not address the role of mitochondrial dysfunction and NADases in these models. Furthermore, the relevance of the autophagy-mitochondria-NAD axis to other cellular fates such as senescence should be tested. Future work should also validate these findings in an *in vivo* setting by carefully monitoring multi-organ dysfunction of autophagy-deficient animals, including the distribution of NAD in different tissues to establish their relative sensitivity to autophagic dysfunction and NAD depletion.

## STAR★METHODS

### RESOURCE AVAILABILITY

#### Lead contact

Further information and requests for resources and reagents should be directed to and will be fulfilled by the lead contact, Viktor I. Korolchuk (viktor.korolchuk@newcastle.ac.uk).

#### Materials availability

Further information and requests for resources and reagents listed in key resources table should be directed to the lead contact.

#### Data and code availability

LC-MS based metabolomics data from this work is available in supplemental information (Table S1). Uncropped immunoblot and RT-PCR images have been deposited at Mendeley and are publicly available as of the date of publication. The DOI is listed in the key resources table. This paper does not report original code. Any additional information required to reanalyse the data reported in this paper is available from the lead contact upon request.

### EXPERIMENTAL MODEL AND SUBJECT DETAILS

#### Culture of mammalian cell lines

*Atg5*^+/+^, *Atg5*^−/−^ MEFs,^6^ Npc1^+/+^ and Npc1^−/−^ MEFs,^42^ wild type and penta-knockout (KO) HeLa cells^23^ were maintained in DMEM supplemented with 10 % fetal bovine serum (FBS), 100 U/mL penicillin/streptomycin and 2 mM L-glutamine at 37 °C, and 5 % CO_2_ in a humidified incubator. HEK293FT cells were cultured as above in medium supplemented with 1X MEM non-essential amino acids. See the key resources table for further information of the mammalian cell lines, medium and reagents used in the study.

#### Culture of human iPSC

Human induced pluripotent stem cells [hiPSCs; control_#13 (WIBR-IPS-NPC1^1920delG/wt^, clone #13), NPC1–1_#4 and #13 (WIBR-IPS-NPC1^I1061T/I1061T^, clones #4 and #13) and NPC1–2_#26 (WIBR-IPS-NPC1^P237S/I1061T^, clone #26) hiPSC lines^35^] were cultured, as previously described.^35,51^ hiPSCs were cultured on a feeder layer of inactivated MEFs in hESC medium consisting of DMEM/F12, 15 % fetal bovine serum (HyClone), 5 % KnockOut Serum Replacement, 1 % L-glutamine, 1 % non-essential amino acids, 1 % penicillin/streptomycin, 4 ng/mL human recombinant basic fibroblast growth factor (bFGF) and 0.1 mM β-mercaptoethanol. For experimentation, the hiPSCs were cultured feeder-free on Geltrex basement membrane matrix in StemFlex Basal Medium supplemented with StemFlex 10X Supplement and 1 % penicillin/streptomycin, or on Matrigel basement membrane matrix in mTeSR1 medium supplemented with mTeSR1 5X supplement and 1 % penicillin/streptomycin. The hiPSCs were maintained on feeders, or feeder-free, in a humidified incubator with 5 % CO_2_ and 5 % O_2_ at 37 °C. There are no ethical requirements for the use of human induced pluripotent stem cells (hiPSCs) in this study because the hiPSC (control and NPC1 patient-derived hiPSCs) lines have been previously published and have undergone many passages in cell culture. The control and NPC1 patient derived hiPSCs were originally generated in the lab of Rudolf Jaenisch at the Whitehead Institute for Biomedical Research. These cell lines were used for this study in the lab of Sovan Sarkar at the University of Birmingham under material transfer agreements, UBMTA 15–0593 and UBMTA 15–0594. All experiments were performed in accordance with ISSCR and institutional guidelines and regulations. See the key resources table for further information of the hiPSCs, medium and reagents used in the study.

#### *Saccharomyces cerevisiae* culture

S288C (*MATα SUC2 gal2 mal2 mel flo1 flo8–1 hap1 ho bio1 bio6*) WT and S288C *atg5Δ::KanMX* were maintained in synthetic complete medium containing 0.13 % drop-out CSM powder, 0.17 % yeast nitrogen base, 2 % glucose, 0.5 % ammonium sulphate. BY4741 (*MATa; his3Δ1 leu2Δ0 ura3Δ0 met15Δ0*) WT and ScPPS2 (BY4741 *atg5Δ*::KanMX) (obtained from Euroscarf), sSUN99 (BY4741 *atg1Δ::Hph*), sRK14 (BY4741 GFP-Atg8:: URA 3), sRK15 (sSUN99 GFP-Atg8:: URA 3) and sRK16 (ScPPS2 GFP-Atg8:: URA 3) (generated in this study) were maintained in synthetic complete medium containing 0.17 % yeast nitrogen base, 2 % glucose, 0.5 % ammonium sulphate, 0.02% histidine, 0.02% methionine, 0,015% lysine, 0.01% leucine, 0.002% uracil, or Ura-dropout medium. Deletion mutants were generated following standard protocols for yeast transformation and genome editing.^52,53^ To generate double deletion mutants, gene deletions were achieved through the replacement of the relevant open-reading frames with the HPHMX resistance gene, amplified from the pAG32 plasmid. Transformants were selected for drug resistance on agar plates and gene deletions were confirmed by PCR on genomic DNA extracts. The strains were grown in the medium to mid-log phase (OD_600_ 0.8–1: S288C WT and *atg5Δ* strains or OD_600_ 0.6 to 0.8: BY4741, ScPPS2, sSUN99, sRK14, sRK15 and sRK16 strains) at 30 C, then washed in dH_2_O twice and switched to a nitrogen starvation medium (SD-N medium: 0.17 % yeast nitrogen base, 2 % glucose) alone or supplemented every 48 h with 10 mM NAM. For immunoblotting and NAD(H) measurement, cultures were collected at the indicated time-points by snap freezing in liquid nitrogen. See the key resources table for further information of the yeast strains, medium and reagents used in the study.

### METHOD DETAILS

#### Differentiation of hiPSCs into neural precursors and human neurons

Differentiation of hiPSCs into neural precursor cells (NPs) and terminally differentiated human neurons were performed as previously described.^35,54^ hiPSC colonies were collected using 1.5 mg/mL collagenase type IV, separated from the MEF feeder cells by gravity, and cultured in non-adherent suspension culture dishes (Corning) in NP medium (NPM) comprising of DMEM/F12 supplemented with 2 % B27, 1 % L-glutamine, 1 % nonessential amino acids and 1 % penicillin/streptomycin supplemented with 500 ng/mL human recombinant Noggin and 10 μM SB431542 for the first 4 days. NPM supplemented with 500 ng/mL Noggin and 20 ng/mL bFGF was used sequentially in the next 2 days (days 6–7), and NPM supplemented with only 20 ng/mL bFGF were used sequentially in the following 7 days (days 7–14) for further NP differentiation. At day 14, NPs clusters were dissociated and plated onto 100 μg/mL poly-L-ornithine and 14 μg/mL laminin pre-coated culture dishes in N2–B27 medium comprising of DMEM/F12 supplemented with 1 % N2, 2 % B27, 1 % L-glutamine, 1 % nonessential amino acids and 1 % penicillin/streptomycin supplemented with 20 ng/mL bFGF and 20 ng/mL EGF. After 7 days in culture, neural rosette-bearing cultures were dissociated using StemPro Accutase and subsequently expanded on poly-L-ornithine and laminin coated cell culture dishes at the density of ~1.5×10^6^ cells per well (of 6-well plate) in N2–B27 medium supplemented with 20 ng/mL bFGF and 20 ng/mL EGF. Proliferating NPs were passaged up to 4 times before induction of terminal differentiation into neurons by growth factor withdrawal in N2–B27 medium. Differentiated neurons were used for analysis 3–4 weeks after differentiation. Supplementation of 1 mM nicotinamide (NAM) was done in hiPSC-derived neurons for the last 6 days (with replenishment on third day) of the neuronal differentiation period (3–4 weeks). See the key resources table for further information of medium and reagents used for neuronal differentiation.

#### Generation of knockout mammalian cell lines using CRISPR/Cas9 gene editing

*Atg5*^−/−^, *Atg7*^−/−^, *Rb1cc1*^−/−^ and Pink1^−/−^ MEFs were generated using the clustered regularly interspaced short palindromic repeats CRISPR/Cas9 system. Ensembl, Aceview and CHOPCHOP databases were utilized to design CRISPR guide RNAs (gRNAs) to target exons present in all splicing variants of the targeted gene. The gRNA oligomer was then annealed and ligated into Bbsl (Fisher Scientific) linearized pSpCas9(BB)-2A-GFP gRNA vector. WT MEFs seeded into a 6-well plate was then used for transfection with DNA ligation products. Cells were allowed to grow for 24 h post seeding before transfection with Lipofectamine^®^ 2000 with 1.6 mg plasmid DNA according to manufacturer’s instructions. GFP-positive cells were sorted by FACS into 96-well plates and expanded into colonies prior to screening by immunoblotting or RT-PCR. See Table S2 for the sequences of sgRNA.

#### Generation of stable mammalian cell lines

Re-introduction of the *Atg5* gene into *Atg5*^−/−^ MEFs and generation of cells stably expressing mRFP-GFP-LC3 (tfLC3), mt-mKeima or NDI1, were achieved by packaging retroviruses or lentiviruses in the HEK293FT (293FT) cell line. 293FT cells were seeded in a 10 cm dish (6.0×10^6^ cells/10 mL/dish) in antibiotic-free glucose culture medium. Next day, cells were transfected with plasmids containing the packaging gag/pol or psPAX2 and envelope pCMV-VSV-G genes, and the pMXs-IP-eGFP-mAtg5, pCHAC-mt-mKeima, pWPIGFP, pWPI-GFP-NDI1 or pSin-TRE-GW-3xHa-puroR-mRFP-GFP-LC3 constructs with Lipofectamine ^®^2000 transfection reagent. Following overnight transfection, the medium was replaced with fresh antibiotic-free medium that was collected after 24 h. Virus containing medium was filtered through at 0.45 μm pore-size filter and overlaid on 30 % confluent cells for 24 h in the presence of 10 μg/mL polybrene. Cells stably expressing the *Atg5* gene or tfLC3 were optimized for protein expression via 2 μg/mL puromycin selection for 7 days. See the key resources table for further information of plasmids used in the study.

#### siRNA transfection

Knockdown of mouse *Parp1*, *Sirt1* was performed by siRNA transfection. MEFs were seeded in a 6-well plate, cultured for 24 h and then transfected with 100 nM siRNA with the Lipofectamine 2000 transfection reagent for 24 h. Cells were then passaged and cultured for 24 h followed by second transfection with 20 nM siRNA for another 24 h prior to galactose culture. See Table S3 for further information of siRNAs used in the study.

#### Galactose medium culture and supplementation in mammalian cells

To induce mitochondrial respiration, cells seeded in a 6-well format (0.3×10^6^ cells/2 mL/well) were switched to a galactose medium (glucose-free DMEM supplemented with 10 mM D-galactose, 10 mM HEPES, 1 mM sodium pyruvate, 4 mM L-glutamine, 100 U/mL penicillin/streptomycin and 10 % FBS) 24 h post-seeding. Galactose medium was supplemented with various compounds and inhibitors: 10–25 mM glucose, 50 μM UK-5099, 10 mM sodium pyruvate, 20 μM Z-VAD-fmk, 10 nM FK866, 20 μM sirtinol, 10 μM olaparib, 1–5 mM NAM, 1 mM NR, 10 mM L-tryptophan, 1 μM rotenone, 5 μM CP2,^25^ 500 nM S1QEL2.2, 100 nM MitoQ, 5 mM N-acetyl-L-cysteine (NAC), 10 mM dimethyl succinate, 1 nM oligomycin, 1 mM adenosine, 1 mM guanosine, 10 mM cytidine, 10 mM uridine or 10 mM thymidine. All compound supplements were added at 0 h, except Z-VAD-fmk which was supplemented at 20 h. For hypoxia experiments, cells were incubated at 1 % O_2_ in an *in vivo* 400 hypoxia work station (Ruskin, UK). Cells were lysed for protein extracts in the chamber to avoid re-oxygenation. See the key resources table for further information of reagents and chemicals used in the study.

#### LC-MS-based metabolomics

Metabolite extraction for liquid-chromatography-mass spectroscopy (LC-MS) was performed, as previously described,^55^ on MEFs following a 16 h incubation in galactose medium. Cells were washed once with cold PBS (Cell Signaling Technology) and lysed at a concentration of 2×10^6^ cells/mL in a metabolite extraction buffer (50 % methanol (Fisher Scientific), 30 % acetonitrile (Sigma-Aldrich), 20 % dH_2_O). Samples were vortexed for 45 s, centrifuged at 16,100 *g* and supernatants subjected to LC-MS as follows, using a three-point calibration curve with universally labelled carbon-13/nitrogen-15 amino acids for quantification. Prepared samples were analysed on a LC-MS platform consisting of an Accela 600 LC system and an Exactive mass spectrometer (Thermo Scientific). A Sequant ZIC-pHILIC column (4.6 mm × 150 mm, 5 μm) (Merck) was used to separate the metabolites with the mobile phase mixed by A=20 mM ammonium carbonate in water and B=acetonitrile. A gradient program starting at 20 % of A and linearly increasing to 80 % at 30 min was used followed by washing (92 % of A for 5 min) and re-equilibration (20 % of A for 10 min) steps. The total run time of the method was 45 min. The LC stream was desolvated and ionised in the HESI probe. The Exactive mass spectrometer was operated in full scan mode over a mass range of 70–1,200 m/z at a resolution of 50,000 with polarity switching. The LC-MS raw data was converted into mzML files by using ProteoWizard and imported to MZMine 2.10 for peak extraction and sample alignment. A house-made database integrating KEGG, HMDB and LIPID MAPS was used for the assignment of LC-MS signals by searching the accurate mass and the metabolites used in the manuscript were confirmed by running their commercial standards. Finally, peak areas were used for comparative quantification.

Output from MS-based metabolomics was subjected to statistical analysis by MetaboAnalyst 4.0 (https://www.metaboanalyst.ca/). We first performed a multivariate statistical principal component analysis (PCA). The variables were normalized by auto-scaling (mean-centered and divided by SD of each variable) by the MetaboAnalyst platform and then subjected to PCA analysis. Furthermore, a univariate statistical test coupled with fold change of each metabolite were plotted on a volcano plot. The significance cut-off was set to an adjusted *P*-value of 0.05 (−Log_10_(*P*-adjusted)>1,3) and a 2 fold-change (−1≥Log_2_(FC)≥1). 1.4-fold-change (−0.51≥Log_2_(FC) ≥0.49 cut-off was applied for the analyses of differences between *Atg5*^−/−^ and *Atg5*^−/−^+NAM. Statistical significance was determined using the Student’s *t*-test with *P* value corrected with the original false discovery rate (FDR) method of Benjamini and Hochberg which does not assume a consistent standard deviation (SD).

#### Immunoblot analysis

##### Mammalian cells

Immunoblotting on cells was performed as described previously.^56^ Cells were washed in ice-cold 1× PBS then lysed for 10 min in RIPA buffer (Sigma-Aldrich) supplemented with Halt Protease & Phosphatase inhibitor cocktail (Thermo Fisher Scientific). Cell lysates were then centrifuged at 4 °C at 16,100 *g* for 10 min to remove insoluble cellular components. Protein concentration was measured using DC Protein Assay (BioRad) and a GloMax plate-reader (Promega). Samples were prepared by boiling with 4xLaemmli sample buffer (BioRad) at 100 °C for 5 min in the presence of 2.5% β-mercaptoethanol (Sigma-Aldrich). Equal amounts of protein (20–40 μg) were subjected to SDS-PAGE with 8–12% Tris-Glycine SDS-PAGE gels and transferred to Immobilon-P PVDF membranes (Millipore) using a TransBlot SD Semi-Dry Transfer Cell (BioRad).

##### hiPSC-derived neurons

Cell lysates were subjected to immunoblot analysis, as previously described.^17,35,57^ Cell pellets were lysed on ice in 2X Lysis Buffer comprising of 20 mM Tris-HCl pH 6.8, 137 mM NaCl, 1 mM EGTA, 1 % Triton X-100, 10 % glycerol and 25X Protease Inhibitor Cocktail (all from Sigma-Aldrich) (buffer made to 1X) for 30 min, boiled for 5–10 min at 100 °C. Protein concentration of the lysates was measured by Bio-Rad Protein Assay (Bio-Rad), and equal amounts of protein (15–40 μg) per sample were subjected to SDS–PAGE.

##### S. cerevisiae

Yeast sample preparation for immunoblotting based on a TCA protein extraction protocol. 10 mL cultures were grown in the appropriate medium to an OD_600_ of 0.8. Cells were pelleted by centrifugation and washed with 20 % TCA (Sigma-Aldrich). All of the following purification steps were performed on ice with pre-chilled solutions. Cell pellets were re-suspended in 100 μL of 20 % TCA and subjected to glass bead lysis. The supernatant was collected, 200 μL of 5 % TCA was added, and the precipitated proteins were pelleted by centrifugation. Protein pellets were solubilized in 30 μL of 2 M Tris pH 8.0 (Sigma-Aldrich) / 70 μL 3X SDS-PAGE loading buffer [60 mM Tris pH 6.8 (Sigma-Aldrich), 2 % SDS (Bio-Rad), 10 % glycerol (Sigma-Aldrich), 100 mM DTT (Sigma-Aldrich), 0.2 % bromophenol blue (Sigma-Aldrich)] and boiled at 95 °C for 5 min. Insoluble material was removed by centrifugation and the supernatant was subjected to SDS-PAGE.

Membranes were first blocked in 5 % milk (Marvel) in PBS with 1x Tween^®^ 20 (Sigma-Aldrich) for 1 h at room temperature and incubated with primary antibodies overnight at 4 °C on a shaker platform. All listed primary antibodies were used at 1:1,000 except β-actin (1:10,000), cleaved caspase-3 (1:200), GAPDH (1:5,000), GFP (1:2,000), LC3B (for hiPSC-derived neurons, 1:2,000), p62 (for MEFs, 1:2,000) and γH2AX (1:2,000) antibodies. Secondary antibodies conjugated to horseradish peroxidase (HRP) were used at 1:5,000 or 1:10,000 dilution for 1 h at room temperature. In mammalian cell samples, clarity western ECL substrate (Bio-Rad Laboratories) was used to visualise chemiluminescence on LAS4000 (Fujifilm). The chemiluminescent signal in yeast samples was generated by the SuperSignal West Pico Plus chemiluminescent substrate (Thermo Scientific) and detected on a G-box transilluminator (Syngene). In hiPSC-derived cell samples, chemiluminescent signal was visualized using Amersham ECL or ECL Prime Western Blotting Detection Reagent and Amersham Hyperfilm ECL (GE Healthcare) via ECOMAX X-ray Film Processor (PROTEC). Densitometry analyses of immunoblots were done using Fiji (ver 1.53c) software. The data were expressed as a ratio or percentage of the control condition, as previously described.^17,35,56,57^ See the key resources table for further information of antibodies used for immunoblot analysis.

#### RNA extraction and RT-PCR

Total RNA was extracted from CRISPR WT or Pink1^−/−^ MEFs using the RNeasy Plus Mini Kit (Qiagen). From 2 mg of total RNA, first-strand complementary DNA (cDNA) was produced using SuperScript III reverse transcriptase (Invitrogen) according to the manufacturer’s instructions. The cDNA was subjected to PCR with OneTaq Hot Start DNA Polymerase (New England Biolabs) and primers listed in Table S4. The amplified products were separated by electrophoresis on 1.5% agarose gel and visualized by SafeView (NBS Biologicals) staining and GelDoc XR+ (BioRad) system.

#### Immunofluorescence

Immunofluorescence analysis was performed on mammalian cells as described previously.^17,56,57^ Cells were washed once with 1x PBS. Cells were fixed in 4 % formaldehyde in PBS for 15 min at room temperature and permeabilized with 0.5 % Triton X-100 for 10 min at room temperature. Cells were then incubated with blocking buffer (5 % goat serum (Sigma-Aldrich) in 1x PBS with 0.05 % Tween-20 for 1 h at room temperature and incubated with primary antibodies overnight at 4 °C. Primary antibodies were used at 1:5,000 (γH2AX) or 1:200 (MAP2 and TUJ1). Cells were washed and incubated with appropriate Alexa Fluor conjugated secondary antibodies at 1:1,000 for 1 h at room temperature. Coverslips were mounted on slides with ProLong^™^ Gold antifade reagent with DAPI. See the key resources table for further information of antibodies used for immunofluorescence.

#### Image acquisition of fixed cells

Fluorescence images of fixed cells were acquired using EVOS FL Cell Imaging System (Thermo Fisher Scientific) with AMG 10x Plan FL and AMG 40x Plan FL lens; Leica DM6000 B (Leica Microsystems) with Leica DFC 350 FX R2 camera and with HC PL APO 40x/1.25 or HC PL APO 100x/1.40 oil immersion lens with Leica Application Suite X software (Leica Microsystems); Axio observer Z1 microscope (Zeiss) with a Plan-Apochromat 20x/0.8 M27 air immersion objective equipped with an Axiocam 503 camera (Zeiss); or with Perkin Elmer UltraView spinning disk confocal system (Perkin Elmer) with an Orca-ER cooled–CCD camera (Hamamatsu) on a Zeiss Axiovert 200 (Carl Zeiss Inc.) with 63× 1.4NA plan-apochromat oil immersion lens using Volocity v6.1 software (Improvision).

#### DNA damage analysis by γH2AX

MEFs were immunostained with γH2AX antibody, and the frequency of γH2AX puncta was assessed by automatic counting using a custom-made plugin in Fiji (ver 1.53c). Quantification was performed on at least 40 cells per condition.

#### Electron microscopy

MEFs seeded in a 6-well format (0.3×10^6^ cells/2 mL/well) were either re-fed glucose medium or switched to a galactose medium 24 h post-seeding. Following a 20 h incubation, cells were trypsinised, washed, collected and fixed overnight in 2 % glutaraldehyde in 0.1 M cacodylate buffer. After rinsing in buffer, the cells were post-fixed in 1 % osmium tetroxide + 1.5 % potassium ferricyanide, rinsed in deionized water then dehydrated through a graded series of acetone. Cells were infiltrated with epoxy resin (TAAB medium) and polymerized at 60 °C for 36 h. Ultrathin sections (70 nm) were picked up on copper grids and stained with uranyl acetate and lead citrate before being viewed on a 100kV CM100 TEM (FEI). Images of 10 cells per cell line were collected and quantified. Mitochondrial morphology of all organelles in a slice was scored in electron microscopy images as either ‘normal’ or ‘abnormal’ and expressed as a ratio of mitochondria with ‘abnormal’ morphology per cell.

#### Separation of sub-cellular fractions in mammalian cells

Mitochondria were isolated from a total of ~60 million (30×6-well) cells by manual cell homogenization in a specialized buffer (20 mM HEPES (pH 7.6) (Sigma-Aldrich), 220 mM D-mannitol (Sigma-Aldrich), 70 mM sucrose (Sigma-Aldrich), 1 mM EDTA (Sigma-Aldrich), 0.5 mM phenylmethylsulfonyl fluoride (Sigma-Aldrich) and 2 mM dithiothreitol (DTT) (Supelco)). Cell homogenates were centrifuged thrice at 800 *g* at 4 °C for 5 min to pellet cellular nuclei and membrane debris. Cytoplasmic and mitochondrial fractions were separated by centrifugation at 16,000 *g* at 4 °C for 10 min and immediately processed for NAD^+^ and NADH measurements, BN-PAGE or immunoblotting. Nuclear fractionation was performed as reported previously ^58^ with slight modifications. Cells were collected from 1 × 10 cm dish and washed with ice-cold PBS. Cells were then suspended in 900 μL of 0.3 % ice-cold NP40 (Sigma-Aldrich) and 300 μL of the lysate was removed as whole cell lysate. The remaining 600 μL lysate was centrifuged for 10 sec in a tabletop centrifuge (ExtraGene mini centrifuge). The supernatant was removed as cytosolic fraction. The pellet was resuspended with 1 mL 0.3 % ice-cold NP40 and centrifuged for 10 sec by the tabletop centrifuge. The supernatant was discarded, and this step was repeated two more times. The pellet was diluted with 180 μL of 1x Laemmli sample buffer and sonicated for extracting nuclear fraction proteins.

#### BN-PAGE

Mitochondria isolated from MEFs were processed for BN-PAGE and immunoblotting as published.^59^ Isolated mitochondria (30 mg) were pelleted, re-suspended in solubilisation buffer [20 mM BisTris pH 7.4 (Santa Cruz Biotechnology), 50 mM NaCl, 10 % glycerol, 10 mM DTT (Supelco)] supplemented with either 1 % triton X-100 (TX-100, Sigma-Aldrich) or 1 % digitonin (Dig, Santa Cruz Biotechnology) and incubated on ice for 20 min. Insoluble material was pelleted by centrifugation at 16,000 *g* for 10 min at 4 °C. The supernatant was mixed with a loading dye [100 mM BisTris pH 7.0 (Santa Cruz Biotechnology), 50 mM ε-amino n-caproic acid (Calbiochem), 5 % Coomassie blue G250 (Sigma-Aldrich)), resolved on a 4 %−13 % gradient acrylamide BN-PAGE gel [acrylamide (Severn Biotech), 150 mM BisTris pH 7.1, 200 mM ε-amino n-caproic acid, 0.1 % ammonium persulfate (Sigma-Aldrich), 0.13 % N,N,N’,N’-Tetramethylethylenediamine (TEMED)] and transferred onto a PVDF membrane followed by a standard immunoblotting protocol.

#### Seahorse analysis

MEFs seeded into Seahorse XF24 V7 assay plates (0.8×10^4^ cells/well) (Agilent Technologies, 100777–004) were either re-fed glucose or switched to a galactose medium, 24 h post-seeding and 20 h prior to analysis. One hour before the assay, the media was switched to unbuffered respective media (with either glucose or galactose) and the plate was incubated at 37 °C without CO_2_. Oxygen consumption rates (OCR) and extracellular acidification rates (ECAR) were determined using Seahorse XF24 analyzer (Agilent Technologies) in the presence of different respiratory and glycolysis inhibitors which were sequentially added as follows: 1.5 μM oligomycin, 3 μM Carbonyl cyanide-4-(trifluoromethoxy)phenylhydrazone (FCCP), and a mixture of 0.5 μM Rotenone, 2.5 μM Antimycin A and 50 mM 2-deoxyglucose. Cellular energetics for ATP production rates by mitochondrial oxidative phosphorylation and glycolysis were calculated by using the OCR and ECAR^60^ based on the methods described,^61^ taking into account the acidification rates due to mitochondrial CO_2_ production. To measure CI- and CII-linked respiration, cells were permeabilised using Seahorse XF Plasma Membrane Permeabilizer (Agilent Technologies), and OCR was measured in the assay buffer (115 mM KCl, 10 mM KH_2_PO_4_, 2 mM MgCl_2_, 3 mM HEPES, 1 mM EGTA and 0.2% fatty acid-free BSA, pH 7.2, at 37°C) with CI substrates (10 mM pyruvate and 1 mM malate) or CII substrate (4 mM succinate and 0.5 μM rotenone). During analysis, the following compounds were added to test mitochondrial activity and cellular bioenergetics flux: 4 mM ADP, 0.5 μM oligomycin, 2.5 μM FCCP and 2.5 μM antimycin A.

#### High-resolution respirometry assay

Respirometry measurements were performed in permeabilised cells using an Oxygraph-2k system (OROBOROS). MEFs seeded into 6-well cell culture dishes (0.3×10^6^ cells/well) were cultured in galactose media for 20 h. Cells were then trypsinised, and 1×10^6^ cells were re-suspended in O2k media (0.5 mM EGTA, 3 mM MgCl_2_, 60 mM lactobionic acid, 20 mM taurine, 10 mM KH_2_PO_4_, 20 mM HEPES, 110 mM D-sucrose, 1 g/L fatty acid free-BSA). Suspended cells were transferred into Oxygraph-2k chambers, permeabilised with digitonin (10 μg/μl), and sequentially supplemented with a combination of pyruvate (5 mM) and malate (2 mM), ADP (4 mM), and succinate (10 mM). The Oxygraph-2k chambers were left to equilibrate after each compound addition for measurement of O_2_ consumption. All measurements were carried out in 2 ml volume at 37°C.

#### MitoSOX staining

MEFs seeded onto 13 mm coverslips in a 6-well format (0.3×10^6^ cells/2mL/well) were either re-fed glucose medium or switched to a galactose medium 24 h post-seeding. A final concentration of 2.5 μM MitoSOX (Invitrogen) was added directly into culture medium followed by a 10 min incubation at 37 °C in the dark. MEFs were then fixed in 3.7 % formaldehyde for 5 min, washed thrice in 1x PBS and mounted on glass slides using a non-DAPI fluoroshield mounting medium. Fluorescence images of fixed cells were compiled at RT on an inverted DMi8-CS microscope (Leica) with a Plan-Apochromat 40x/1.30 oil immersion objective, equipped with an ORCA-Flash4v2.0 camera (Hamamatsu). MitoSOX staining intensity was analyzed in Fiji (ver 1.53c) by outlining single cells as regions of interest and calculation of the raw integrated density value per cell. Quantification was performed on 30–40 cells per condition.

#### Mitophagy measurement

Cells stably expressing mt-mKeima were grown in a 96-well glass bottom plate (Greiner Bio-One) (0.55×10^4^ /100 μL/well, 24 h) and cultured in the indicated conditions. The live-cell mt-mKeima signal was acquired sequentially using a Leica DMi8 inverted microscope with a Plan-Apochromat 63x/1.30 oil immersion objective, equipped with an ORCA-Flash4v2.0 camera (Hamamatsu). Images were deconvolved using Huygens Essential software (ver 20.10; Scientific Volume Imaging). Mitophagy events were determined as the number of puncta per cell in images generated by subtracting the signal at a 480 nm excitation (reporting a neutral pH-environment) from the signal at a 561 nm excitation (reporting an acidic pH-environment), by using Fiji (ver 1.53c).

#### Mitochondrial ΔΨm measurement

For measuring mitochondrial ΔΨm in MEFs, cells were grown in a 96-well glass bottom plate (Greiner Bio-One) (0.8×10^4^ /100 μL/well, 24 h). Following culture in galactose media for 20 h, cells were co-stained with 16.7 nM tetramethylrhodamine methyl ester (TMRM) and 100 nM Mitotracker Green (MTG) or stained with TMRM alone. A 10x stock of each compound was prepared in conditioned galactose medium (24 h culture on *Atg5*^−/−^ MEFs, collected and filtered through a 0.22 mm pore-size filter) and added directly to culture wells. Following a 30 min incubation in the dark at 37 °C, TMRM- and MTG-containing medium was replaced by dye-free conditioned galactose medium. Live cell imaging was performed in a maintained atmosphere of 37 °C and 5 % CO_2_ using an LSM700 microscope (Zeiss) with a C-Apochromat 40x/1.20 water immersion lens, capturing images line sequentially. Images were deconvolved using Huygens Essential software (ver 20.10; Scientific Volume Imaging). TMRM and MTG raw integrated density values per cell were quantified using Fiji (ver 1.53c) by outlining single cells as regions of interest. Mitochondrial ΔΨm was expressed as a ratio of TMRM to MTG, or relative intensity of TMRM. Quantification was performed on at least 30 cells per condition. See the key resources table for further information of reagents used for mitochondrial ΔΨm measurement.

#### NAD^+^ and NADH measurements

Measurements of NAD^+^ and NADH in mammalian whole cell lysates, mitochondrial lysates, and in yeast cells were performed as described in a published protocol.^62^ NAD^+^ or NADH were extracted with an acidic solution (10 % (mammalian cells and isolated mitochondria) or 20 % (yeast) trichloroacetic acid (TCA) or a basic solution (0.5 M sodium hydroxide (NaOH), 5 mM EDTA), respectively. NAD^+^ and NADH pools from cellular cytoplasmic fractions were extracted by addition of 5x concentrated stocks of TCA and NaOH/EDTA solutions into the cytoplasmic supernatant. Samples were adjusted to pH 8 with 1 M Tris (Sigma-Aldrich). NAD^+^ and NADH levels were determined by fluorescence intensity of resorufin produced by an enzymatic cycling reaction using resazurin, riboflavin 5’-monophosphate, alcohol dehydrogenase and diaphorase. Fluorescence intensity was monitored every minute for a total 60 min using a microplate reader (FLUOstar Omega, BMG Labtech). NAD^+^ and NADH levels were determined by a β-NAD standard curve and adjusted to protein concentration determined by the DC protein assay.

Measurements of NAD^+^ and NADH in hiPSC-derived neurons were done using NAD/NADH Quantitation Colorimetric Kit (BioVision) according to manufacturer’s instructions. Control and NPC1 neurons were washed with cold PBS, then lysed with NADH/NAD Extraction Buffer and immediately freeze-thawed twice on dry ice, then centrifuged at 14,000 rpm for 5 min at 4 °C. Half of the supernatant was incubated at 60 °C for 30 min to decompose the NAD and detect the NADH. Both halves of the supernatants were then cooled on ice, transferred into a 96-well plate, followed by incubation in Reaction Mix comprising of NADCycling Buffer and NAD Cycling Enzyme Mix for 5 min at room temperature. Then NADH Developer was added to each well and the reaction was left to cycle for 1–2 h at room temperature. Measurements of optical density (OD) at 450 nm using the EnSpire Multimode plate reader (Perkin Elmer) were performed every 20–30 min to detect the saturating OD, then normalized to protein concentration via Bio-Rad Protein Assay to measure pmol/mg of NAD^+^ and NADH. See the key resources table for further information of reagents used for NAD^+^ and NADH measurements.

#### Assessment of autophagy

##### Mammalian cells

Cells stably expressing tfLC3 were grown in a 96-well glass bottom plate (Greiner Bio-One) (0.55×10^4^ /100 μL/well, 24 h). Following culture in glu or gal media for 96 h, the media were replaced by dye-free medium. Live cell imaging was performed in a maintained atmosphere of 37 °C and 5 % CO_2_ using an LSM700 microscope (Zeiss) with a C-Apochromat 63x/1.40 oil immersion lens, capturing images line sequentially. Images were deconvolved using Huygens Essential software (ver 20.10; Scientific Volume Imaging). The number of autophagosomes (GFP^+^ RFP^+^ puncta) and autolysosomes (GFP^−^ RFP^+^ puncta) per cell were quantified using Fiji (ver 1.53c) by outlining single cells as regions of interest. Quantification was performed on at least 30 cells per condition.

##### hiPSC-derived neurons

Autophagic activity was assessed by the degradation of autophagy marker proteins, p62 and LC3, by immnoblot analysis.

##### S. cerevisiae

Autophagic activity in S288C WT and *atg5Δ* strains was assessed upon transformation of the strains with a GFP-ATG8(416)/GFP-AUT7(416) plasmid. The transformed strains were switched to SD-N media and collected prior to the switch (0 h) and at 2 h, 4 h and 18 h time-points following the switch. Samples equivalent to 5 mL at OD_600_ 1, were taken at the indicated times for protein extraction and immunoblot analysis. Autophagic activity in BY4741, sSUN99 and ScPPS2 strains was assessed upon transformation of the strains with pRS316-GFP-Atg8 plasmid. The strains (sRK14, sRK15 and sRK16) were switched to SD-N media and collected at 4 h. Cells were stained with 3 μM CellTracker Blue CMAC Dye for 30 min at 30 °C in dark and mounted on 2% agarose beds on glass slides. Images were obtained using a DeltaVision microscope (GE Healthcare) with a 60x objective, a cool-SNAP HQ camera and softWoRx software (GE Healthcare), and quantified using Fiji (ver 1.53c). Representative images were deconvolved using Huygens Essential software (ver 20.10; Scientific Volume Imaging). See the key resources table for further information of plasmids used for the assessment of autophagy in yeast.

#### Cell death assays in mammalian cells

Adherent and floating cells were collected and processed by protein extraction and immunoblot analysis of caspase-3 cleavage at 24 h (*Atg5*^+/+^ and Atg5^−/−^ MEFs), 72 h (Npc1^+/+^ and Npc1^−/−^) and 110 h (CRISPR/Cas9 generated control, Atg5^−/−^, Atg7^−/−^ and Rb1cc1^−/−^ cell lines) after media switch. Representative phase-contrast images and fluorescence images stained with ReadyProbes Cell Viability Imaging Kit (Invitrogen) were obtained on an inverted DM-IL Leica microscope equipped with an Invenio 3SII digital camera (3.0 Mpix Colour CMOS; Indigo Scientific).

#### Cytotoxicity assay in mammalian cells

Cytotoxicity was measured using CytoTox-Glo Cytotoxicity Assay (Promega) according to manufacturer instruction. This luminescence-based cytotoxicity assay measures the extracellular activity of a distinct dead-cell protease when it is released from membrane-compromised cells. Cells in 96-well plates cultured for the indicated times after media switch were incubated with CytoTox-Glo Assay Reagent (comprising of Assay Buffer and AAF-Glo Substrate) for 15 min at room temperature in the dark, then luminescence was measured using GloMax plate-reader (Promega) and the readings obtained were attributed to the basal cytotoxicity per well (first reading). To estimate cell population per well, cells were further incubated with Lysis Reagent (comprising of Assay Buffer and Digitonin) for 30 min at room temperature in the dark, after which luminescence was measured again (second reading). Cytotoxicity data were normalized by dividing the first reading (basal cytotoxicity per well) to the second reading (indicative of cell population per well) and expressed as a percentage.

#### Cell viability assays and growth curve analysis in yeast cells

##### Spot-test assay

Cultures were processed for a spot-test assay 5–7 days after the switch to SD-N medium. The individual strain concentrations were equalised by OD600 and subsequently spotted on to YEPD plates (YEPD broth (1 % yeast extract, 2 % peptone, 2 % glucose, 2 % agar) in a 5-fold serial dilution and left to grow at 30 °C for 48 h prior to imaging in G-box transilluminator (Syngene).

##### Phloxine B staining

Cells collected on day 0, 2, 4, 5, 6, and 7 after the switch to SD-N medium were pelleted, resuspended in fresh media containing 2 μg/mL Phloxine B (See the key resources table), and mixed by gently inverting 3 times. Cells were then washed in dH_2_O twice and mounted on 2% agarose beds. Images were obtained using a DeltaVision microscope (GE Healthcare) with a 60x objective, a cool-SNAP HQ camera and softWoRx software (GE Healthcare) and quantified using Fiji (ver 1.53c).

##### Growth curve analysis

Cells seeded in a 96-well plate were cultured in complete medium or SD-N medium supplemented with or without 10 mM NAM at 30 °C with shaking (180 spm). OD_600_ was recorded every 2 h up to 48 h by using a Varioscan Flash (Thermo Scientific) plate reader.

#### TUNEL staining for apoptotic neuronal cells

Neurons differentiated from hiPSCs were stained with Click-iT Plus TUNEL Assay (See the key resources table) for in situ apoptosis detection, Alexa Fluor 488 dye (Invitrogen), according to the manufacturer’s protocol. hiPSC-derived neurons were fixed with 4 % formaldehyde (Thermo Fisher Scientific) for 15 min and permeabilised with 0.25 % Triton X-100 (Sigma-Aldrich) for 20 min at room temperature, then washed with deionized water. Cells were incubated at 37 °C for 10 min in TdT reaction buffer, followed by incubation with TdT reaction mixture containing TdT reaction buffer, EdUTP, TdT enzyme for 60 min at 37 °C, washed with 3 % BSA, and finally incubated with Click-iT Plus TUNEL reaction cocktail for 30 min at 37 °C followed by washes with 3 % BSA. For detection of TUNEL^+^ apoptotic nuclei specifically in neurons, cells were subjected to immunofluorescence by blocking with 3 % BSA (in PBS) followed by incubation with anti-TUJ1 antibody (in 3% BSA in PBS) overnight at 4 °C, and thereafter incubated with Alexa Fluor 594 secondary antibody for 1 h at room temperature. Coverslips were mounted on glass slides with ProLong Gold antifade reagent with DAPI (Invitrogen). The quantification of TUNEL^+^ apoptotic nuclei in TUJ1^+^ neuronal cells was performed via fluorescence microscopy, as previously described.^35^ The percentage of TUNEL^+^ nuclei was calculated from the total number of TUJ1^+^ cells analyzed (~200–300 cells per sample were analysed).

### QUANTIFICATION AND STATISTICAL ANALYSIS

All experiments were carried out in three or more biological replicates. Quantifications of data and statistical analysis of metabolomics data are described under various Method details sections where applicable. Graphical data denote the mean ± s.e.m (of *n* = 3 or more biological replicates) and are depicted by column graph scatter dot plot, or displayed as cell popular violin plots, using Prism 8.3.1 software (GraphPad). Unless indicated otherwise, the *P* values for analyses was determined by Student’s *t* test (two-tailed, unpaired) between two groups or one-way ANOVA followed by multiple comparisons with the two-stage linear step-up procedure of Benjamini, Krieger and Yekutieli (with an FDR value of 0.05) using Prism 8.4.3 software (GraphPad). * or ^§^, *p*<0.05; **, *p*<0.01; ***, *p*<0.001; ns (non-significant).

## Supplementary Material

Supplementary Table S1

1

## Figures and Tables

**KEY RESOURCES TABLE:** 

Reagent or resource	Source	Identifier

Antibodies		

α-tubulin	Cell Signaling Technology	Cat#2144; RRID: AB_2210548
β-actin	Cell Signaling Technology	Cat#4970; RRID: AB_2223172
Acetylated lysine (AcK)	Cell Signaling Technology	Cat#9441; RRID: AB_331805
AIF	Cell Signaling Technology	Cat#5318; RRID: AB_10634755
ATG5	Sigma-Aldrich	Cat#A0856; RRID: AB_1078238
Cleaved caspase-3^(Asp175)^	Cell Signaling Technology	Cat#9661; RRID: AB_2341188
GAPDH	Sigma-Aldrich	Cat#G8795; RRID: AB_1078991
GFP (for MEFs)	Santa Cruz Biotechnology	Cat#sc-9996; RRID: AB_627695
GFP (for yeast)	Roche	Cat#11814460001; RRID: AB_390913
Histone H2A.X (total H2AX)	Cell Signaling Technology	Cat#2595; RRID: AB_10694556
Histone H3	Cell Signaling Technology	Cat#4499; RRID: AB_10544537
LC3B (for MEFs)	Cell Signaling Technology	Cat#3868; RRID: AB_2137707
LC3B (for hiPSC-derived cells)	Novus Biologicals	Cat#NB100–2220; RRID: AB_10003146
MAP2	Thermo Fisher Scientific	Cat#PA5–17646; RRID: AB_11006358
NDI1	Gift from Takao Yagi (Scripps Institute)	N/A
NDUFA9	Abcam	Cat#ab14713; RRID: AB_301431
NDUFS1	Abcam	Cat#ab169540; RRID: AB_2687932
NDUFS3	Abcam	Cat#ab110246; RRID: AB_10861972
p62 (for MEFs)	Progen	Cat#GP62-C; RRID: AB_2687531
p62 (for hiPSC-derived cells)	BD Biosciences	Cat#610832; RRID: AB_398151
pan-ADP-ribose	Millipore	Cat#MABE1016; RRID: AB_2665466
PARP1	Cell Signaling Technology	Cat#9532; RRID: AB_659884
Phospho-Histone H2A.X (γH2AX)	Millipore	Cat#05-636; RRID: AB_309864
Poly(ADP-ribose) (PAR)	Enzo Life Sciences	Cat#ALX-804-220-R100; RRID: AB_2052275
SDHA	Cell Signaling Technology	Cat#11998; RRID: AB_2750900
SIRT1	Millipore	Cat#07-131; RRID: AB_10067921
TUJ1 (TUBB3)	BioLegend	Cat#801201; RRID: AB_2313773
Goat anti-mouse IgG, H&L chain specific peroxidase conjugate	Millipore	Cat#401253; RRID: AB_437779
Goat anti-rabbit IgG, H&L chain specific peroxidase conjugate	Millipore	Cat#401393; RRID: AB_437797
Rabbit anti-guinea pig IgG, peroxidase conjugate	Dako	Cat#P014102; RRID: N/A
Goat anti-mouse IgG (H+L), Alexa Fluor 488	Thermo Fisher Scientific	Cat#A-11001; RRID: AB_2534069
Goat anti-mouse IgG (H+L), Alexa Fluor 594	Thermo Fisher Scientific	Cat#A-11005; RRID: AB_2534073
Goat anti-rabbit IgG (H+L), Alexa Fluor 488	Thermo Fisher Scientific	Cat#A-11008; RRID: AB_143165
Goat anti-rabbit IgG (H+L), Alexa Fluor 594	Thermo Fisher Scientific	Cat#A-11012; RRID: AB_2534079

Chemicals, peptides, and recombinant proteins		

DMEM	Sigma-Aldrich	Cat#D6546
DMEM glucose free	Gibco	Cat#A1443001
Penicillin/streptomycin	Sigma-Aldrich	Cat#P4333
L-Glutamine	Sigma-Aldrich	Cat#G7513
Sodium pyruvate solution	Sigma-Aldrich	Cat#S8636
HEPES solution	Sigma-Aldrich	Cat#H0887
FBS	Sigma-Aldrich	Cat#F0804
1xMEM non-essential amino acids	Sigma-Aldrich	Cat#M7145
DMEM/F12	Gibco	Cat#11320-074
FBS	HyClone	Cat#SH30071.03
KnockOut Serum Replacement	Gibco	Cat#10828-028
L-glutamine	Gibco	Cat#25030-024
Penicillin/streptomycin	Gibco	Cat#15070063
bEGF	Gibco	Cat#PHG0313
β-mercaptoethanol	Sigma-Aldrich	Cat#M3148
Geltrex	Gibco	Cat#A1413302
StemFlex Basal Medium	Gibco	Cat#A33494-01
StemFlex 10X Supplement	Gibco	Cat#A33492-01
Matrigel	Corning	Cat#354277
mTeSR1	Stem Cell technologies	Cat#85850 (bundle)
mTeSR1 5X supplement	Stem Cell technologies	Cat#85850 (bundle)
Collagenase type IV	Gibco	Cat#17104019
B27	Gibco	Cat#12587-010
human recombinant Noggin	Peprotech	Cat#120-10C
SB431542	Stemgent	Cat#04-0010-10
bFGF	R&D Systems	Cat#RP8627
poly-L-ornithine	Sigma-Aldrich	Cat#P4957
Laminin	Sigma-Aldrich	Cat#L2020
N-2 Supplement	Gibco	Cat#17502-048
StemPro Accutase	Gibco	Cat#A1110501
Drop-out CSM powder (for S288C strain)	Formedium	Cat#DCS0351
yeast nitrogen base (for S288C strain)	Formedium	Cat#CYN0401
D-glucose (for S288C strain)	Formedium	Cat#GLU02
Ammonium sulphate (for S288C strain)	Sigma-Aldrich	Cat#A2939
Yeast extract (for S288C strain)	Formedium	Cat#YEA02
Peptone (for S288C strain)	Formedium	Cat#PEP02
Agar (for S288C strain)	Formedium	Cat#AGA02
Yeast nitrogen base (for BY4741 strain)	Himedia	Cat#M151
D-(+)-Glucose (for BY4741 strain)	Merck	Cat#1.94925.5021
Ammonium sulphate (for BY4741 strain)	Merck	Cat#1.93246.0521
L-Histidine (for BY4741 strain)	Himedia	Cat#GRM051
L-Leucine (for BY4741 strain)	Himedia	Cat#GRM054
L-Lysine (for BY4741 strain)	Sigma-Aldrich	Cat#L5501
L-Methionine (for BY4741 strain)	Himedia	Cat#GRM200
Uracil (for BY4741 strain)	Himedia	Cat#GRM264
Antimycin A	Sigma-Aldrich	Cat#A8674; CAS: 1397-94-0
Bafilomycin A1	Enzo Life Sciences	Cat#BML-CM110-0100; CAS: 88899-55-2
CP2	Zhang et al.^25^	N/A
Cytidine	Sigma-Aldrich	Cat#C4654; CAS: 65-46-3
D-(+)-Galactose	Sigma-Aldrich	Cat#G0750; CAS: 59-23-4
D-(+)-Glucose	Sigma-Aldrich	Cat#G5767; CAS: 50-99-7
Dimethyl succinate	Sigma-Aldrich	Cat#W239607; CAS: 106-65-0
FK866	Sigma-Aldrich	Cat#F8557; CAS: 658084-64-1
Guanosine	Sigma-Aldrich	Cat#G6264; CAS: 118-00-3
L-Tryptophan	Sigma-Aldrich	Cat#T0254; CAS: 73-22-3
MitoQ	Gift from Michael Murphy (University of Cambridge)	N/A
N-acetyl-L-cysteine (NAC)	Sigma-Aldrich	Cat#A7250; CAS: 616-91-1
Nicotinamide (NAM)	Sigma-Aldrich	Cat#N0636; CAS: 98-92-0
Nicotinamide riboside (NR)	Provided from ChromaDex	N/A
Olaparib	Cayman Chemical	Cat#10621; CAS: 763113-22-0
Oligomycin	Sigma-Aldrich	Cat#495455; CAS: 1404-19-9
Rotenone	Sigma-Aldrich	Cat#R8875; CAS: 83-79-4
S1QEL2.2	Life Chemicals	Cat#F2068-0013; CAS:951535-81-2
Sirtinol	Cayman Chemical	Cat#10523; CAS: 410536-97-9
Sodium pyruvate	Sigma-Aldrich	Cat#P2256; CAS: 113-24-6
Thymidine	Sigma-Aldrich	Cat#T1895; CAS: 50-89-5
UK-5099	Sigma-Aldrich	Cat#PZ0160; CAS: 56396-35-1
Uridine	Sigma-Aldrich	Cat#U3003; CAS: 58-96-8
Z-VAD-FMK	Enzo Life Sciences	Cat#ALX-260-020-M001; CAS: 220644-02-0
Lipofectamine 2000	Invitrogen	Cat#11668019
Polybrene	Sigma-Aldrich	Cat#107689
Puromycin	Gibco	Cat#A1113803
MitoSOX	Invitrogen	Cat#M36008
MitoTracker Green	Invitrogen	Cat#M7514
Tetramethylrhodamine, Methyl Ester, Perchlorate (TMRM)	Invitrogen	Cat#T668
CellTracker Blue CMAC Dye	Invitrogen	Cat#C2110
Phloxine B	Sigma-Aldrich	Cat#P2759
ProLong Gold Antifade Mountant with DAPI	Invitrogen	Cat#P36931
Anti-Fade Fluorescence Mounting Medium - Aqueous, Fluoroshield	Abcam	Cat#ab104135
Trichloroacetic acid (TCA)	Sigma-Aldrich	Cat#T4885
Sodium hydroxide	Sigma-Aldrich	Cat#S5881
EDTA	Fisher scientific	Cat#BP120-500
Tris	Sigma-Aldrich	Cat#T1503
Resazurin	Sigma-Aldrich	Cat#R7017
Riboflavin 5'-monophosphate	Sigma-Aldrich	Cat#F8399
Alcohol dehydrogenase	Sigma-Aldrich	Cat#A3263
Diaphorase	Sigma-Aldrich	Cat#D5540
β-NAD	Sigma-Aldrich	Cat#N0632
DC protein assay	BioRad	Cat#5000116

Critical commercial assays		

Cytotox-Glo Cytotoxicity Assay	Promega	Cat#G9291
NAD/NADH Quantitation Colorimetric Kit	BioVision	Cat#K337
ReadyProbes Cell Viability Imaging Kit	Invitrogen	Cat#R37609
Click-iT Plus TUNEL Assay for in situ apoptosis detection, Alexa Fluor 488 dye (Invitrogen)	Invitrogen	Cat#C10617

Deposited data		

LC-MS metabolomics data	This paper	Table S1
Uncropped scan of immunoblotting and RT-PCR images	This paper	Mendeley Data:https://doi.org/10.17632/frhjtk3jf9.1

Experimental models: Cell lines		

Atg5^+/+^ MEFs	Gift from Noboru Mizushima^6^ (University of Tokyo)	N/A
Atg5^−/−^ MEFs	Gift from Noboru Mizushima^6^ (University of Tokyo)	N/A
Npc1^+/+^ MEFs	Gift from Peter Lobel^42^ (Rutgers University)	N/A
Npc1^−/−^ MEFs	Gift from Peter Lobel^42^ (Rutgers University)	N/A
CRISPR control MEFs	This paper	N/A
*Atg5*^CRISPR−/−^ MEFs	This paper	N/A
*Atg7*^CRISPR−/−^ MEFs	This paper	N/A
*Rb1cc1*^CRISPR−/−^ MEFs	This paper	N/A
*Pink1*^CRISPR−/−^ MEFs	This paper	N/A
Wild-type HeLa	Lazarou et al.^23^	N/A
PentaKO HeLa	Lazarou et al.^23^	N/A
293FT	Invitrogen	Cat#R70007
WIBR-IPS-NPC1^1920delG/wt^, clone #13	Maetzel et al.^35^	N/A
WIBR-IPS-NPC1^I1061T/I1061T^, clone #4	Maetzel et al.^35^	N/A
WIBR-IPS-NPC1^I1061T/I1061T^, clone #13	Maetzel et al.^35^	N/A
WIBR-IPS-NPC1^P237S/I1061T^, clone #26	Maetzel et al.^35^	N/A

Experimental models: Organisms/strains		

*S. cerevisiae*: S288C (*MAT*α *SUC2 gal2 mal2 mel flo1 flo8-1 hap1 ho bio1 bio6*) WT	Open BioSystems	N/A
*S. cerevisiae*: S288C *atg5Δ::KanMX*	This paper	N/A
*S. cerevisiae*: BY4741 (*MATa; his3Δ1 leu2Δ0 ura3Δ0 met15Δ0*)	Euroscarf	Acc. No. Y00000
*S. cerevisiae*: ScPPS2 (BY4741 *atg5Δ::KanMX*)	Euroscarf	Acc. No. Y02103
*S. cerevisiae*: sSUN99 (BY4741 *atg1Δ:: Hph*)	This paper	N/A
*S. cerevisiae*: sRK14 (BY4741 GFP-Atg8:: URA 3)	This paper	N/A
*S. cerevisiae*: sRK15 (sSUN99 GFP-Atg8:: URA 3)	This paper	N/A
*S. cerevisiae*: sRK16 (ScPPS2 GFP-Atg8:: URA 3)	This paper	N/A

Oligonucleotides		

sgRNA sequences, see Table S2	This paper	N/A
siRNAs, see Table S3	This paper	N/A
Primers for RT-PCR, see Table S4	This paper	N/A

Recombinant DNA		

psPAX2	Gift from Didier Trono (Ecole Polytechnique Fédérale de Lausanne)	Addgene Plasmid, #12260
pCMV-VSV-G	Gift from Bob Weinberg^43^ (Massachusetts Institute of Technology)	Addgene Plasmid, #8454
pMXs-IP-eGFP-mAtg5	Gift from Noboru Mizushima^44^ (University of Tokyo)	Addgene Plasmid, #38196
pCHAC-mt-mKeima	Lazarou et al.^23^	Addgene Plasmid, #72342
pSin-TRE-GW-3xHa-puroR-mRFP-GFP-LC3	Gift from Raphael Roduit^45^ (University of Lausanne)	N/A
pWPI-GFP	Gift from Eric Dufour^46^ (University of Tampere)	N/A
pWPI-GFP-NDI1	Gift from Eric Dufour^46^ (University of Tampere)	N/A
pRS416-GFP-ATG8(416)/GFP-AUT7(416)	Gift from Daniel Klionsky^47^ (University of Michigan)	Addgene Plasmid, #49425
pRS316-GFP-Atg8	Gift from Yoshinori Ohsumi (Tokyo Institute of Technology)	N/A
pSpCas9(BB)-2A-GFP gRNA vector	Gift from Feng Zhang^48^ (Massachusetts Institute of Technology)	Addgene Plasmid, #48138

Software and algorithms		

ImageJ/Fiji (version 1.53c)	Schindelin et al.^49^	http://fiji.sc; RRID: SCR_002285
Prism8	GraphPad	http://www.graphpad.com/; RRID: SCR_002798
Huygens Essential	Scientific Volume Imaging	https://svi.nl/HuygensSoftware; RRID: SCR_014237
MetaboAnalyst	Xia and Wishart^50^	https://www.metaboanalyst.ca/; RRID:SCR_015539

## Highlights

- Loss of autophagy in respiring cells causes a metabolic deficit and cell death
- Mitochondrial dysfunction due to the loss of basal mitophagy underlies cellular stress
- NADases of PARP and Sirtuin families are hyperactive in autophagy-deficient cells
- NAD depletion contributes to death of autophagy-/mitophagy-deficient cells

## INCLUSION AND DIVERSITY

We support inclusive, diverse, and equitable conduct of research.
