# Supplementary material for: Autophagy promotes cell survival by maintaining NAD levels
Source: Dev Cell. Author manuscript; Available in PMC 2024 Oct 15. (PMC11475545; doi:10.1016/j.devcel.2022.10.008)
Supplement: 1 [file NIHMS2017691-supplement-1.pdf]

Developmental Cell, Volume *57*

Supplemental information

Autophagy promotes cell survival by maintaining NAD levels

Tetsushi Kataura, Lucia Sedlackova, Elsje G. Otten, Ruchika Kumari, David Shapira, Filippo Scialo, Rhoda Stefanatos, Kei-ichi Ishikawa, George Kelly, Elena Seranova, Congxin Sun, Dorothea Maetzel, Niall Kenneth, Sergey Trushin, Tong Zhang, Eugenia Trushina, Charles C. Bascom, Ryan Tasseff, Robert J. Isfort, John E. Oblong, Satomi Miwa, Michael Lazarou, Rudolf Jaenisch, Masaya Imoto, Shinji Saiki, Manolis Papamichos-Chronakis, Ravi Manjithaya, Oliver D.K. Maddocks, Alberto Sanz, Sovan Sarkar, and Viktor I. Korolchuk

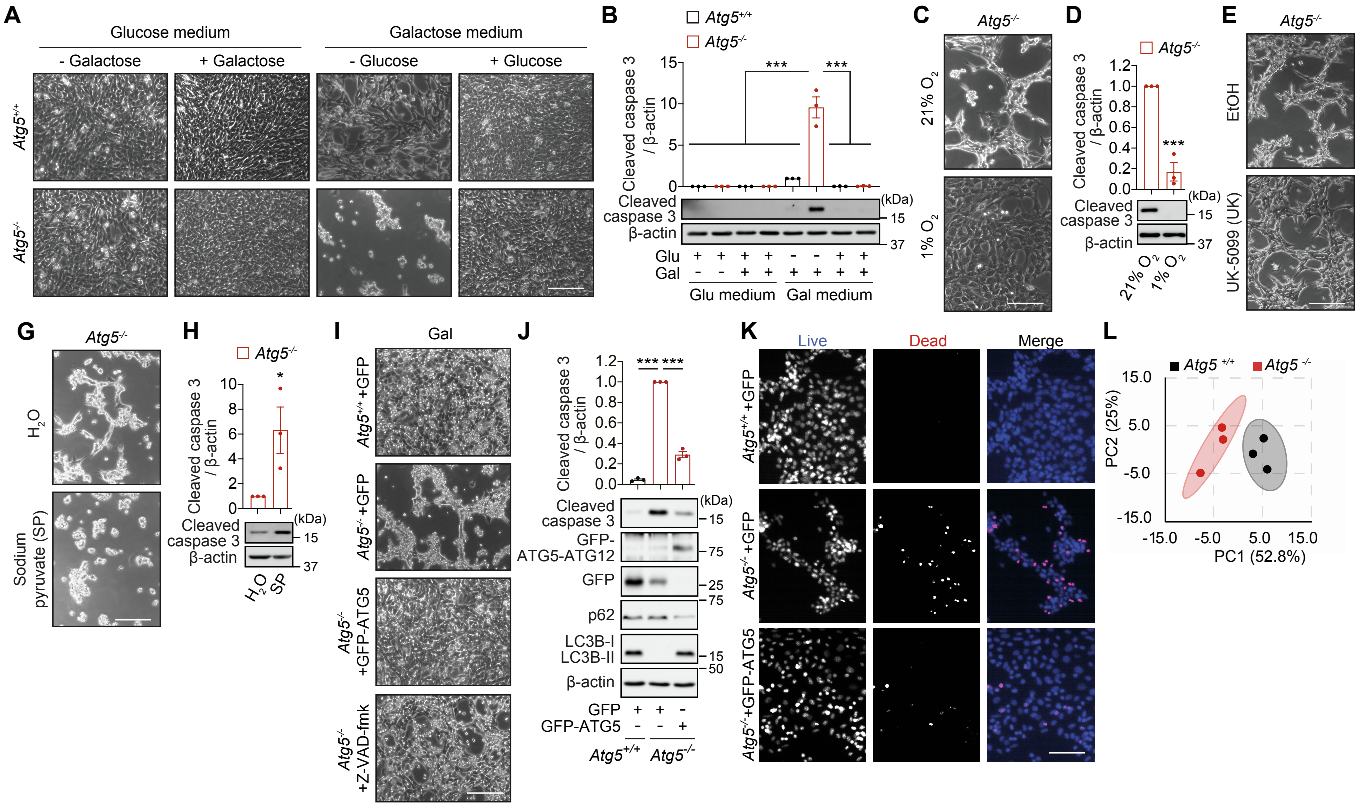

A
Glucose medium
- Galactose
+ Galactose
Galactose medium
- Glucose
+ Glucose
Atg5+/+
Atg5-/-
B
Atg5+/+
Atg5-/-
Cleaved caspase 3 / β-actin
***
***
Cleaved caspase 3
β-actin
(kDa)
15
37
Glu
Gal
Glu medium
Gal medium
C
Atg5-/-
21% O2
1% O2
D
Atg5-/-
Cleaved caspase 3 / β-actin
***
Cleaved caspase 3
β-actin
21% O2
1% O2
E
Atg5-/-
EtOH
UK-5099 (UK)
F
Atg5-/-
Cleaved caspase 3 / β-actin
***
Cleaved caspase 3
β-actin
EtOH
UK5099
G
Atg5-/-
H2O
Sodium pyruvate (SP)
H
Atg5-/-
Cleaved caspase 3 / β-actin
*
Cleaved caspase 3
β-actin
H2O
SP
I
Gal
Atg5+/+ +GFP
Atg5-/- +GFP
Atg5-/- +GFP-ATG5
Atg5-/- +Z-VAD-fmk
J
Cleaved caspase 3 / β-actin
*** ***
Cleaved caspase 3
GFP-ATG5-ATG12
GFP
p62
LC3B-I
LC3B-II
β-actin
GFP
GFP-ATG5
Atg5+/+
Atg5-/-
K
Live
Dead
Merge
Atg5+/+ +GFP
Atg5-/- +GFP
Atg5-/- +GFP-ATG5
L
Atg5 +/+
Atg5 -/-
PC2 (25%)
PC1 (52.8%)

Figure S1. Respiration-dependent apoptotic phenotype in autophagy-deficient cell lines cultured in galactose medium. Related to Figure 1.

A, B, Phase contrast images (A) and immunoblot analyses (B) of *Atg5*$^{+/+}$ or *Atg5*$^{-/-}$ MEFs cultured in glu or gal medium with or without glu or gal for 24 h. **C–H,** Phase contrast images (C, E, G) and immunoblot analyses (D, F, H) of *Atg5*$^{-/-}$ MEFs cultured for 24 h in gal medium in atmospheric oxygen (21 % O_2) or in hypoxia (1 % O_2) (C, D), in the presence or absence of UK-5099 (E, F), or in the presence or absence of 10 mM sodium pyruvate (SP) supplementation (G, H). **I,** Phase contrast images of *Atg5*$^{+/+}$+GFP, *Atg5*$^{-/-}$+GFP, *Atg5*$^{-/-}$+GFP-Atg5 MEFs, or *Atg5*$^{-/-}$ MEFs supplemented with Z-VAD-fmk, cultured in gal medium for 24 h. **J,** Immunoblot analyses of *Atg5*$^{+/+}$+GFP, *Atg5*$^{-/-}$+GFP, *Atg5*$^{-/-}$+GFP-Atg5 MEFs in the same conditions as (J). **K,** Staining for cell death with ReadyProbes fluorescent dyes in *Atg5*$^{+/+}$+GFP, *Atg5*$^{-/-}$+GFP and *Atg5*$^{-/-}$+GFP-Atg5 MEFs 24 h after switch to gal medium. **L,** Two-dimensional principal component analysis (PCA) scores plot of metabolites in *Atg5*$^{+/+}$ (black) vs *Atg5*$^{-/-}$ (red) MEFs cultured in gal medium. Graphical data are mean ± s.e.m of n = 3 biological replicates (B, D, F, H, J). P values were calculated by unpaired two-tailed Student's t-test (D, F, H) or one-way ANOVA followed by multiple comparisons with the two-stage linear step-up procedure of Benjamini, Krieger and Yekutieli (B, J) on three independent experiments. *$P<0.05$; ***$P<0.001$. Scale bars, 200 µm (A, C, E, G, I, K).

A B

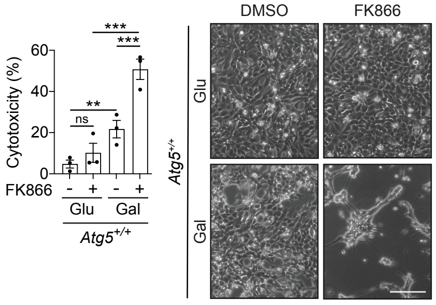

C

Positive change-discovered metabolites (*Atg5*$^{-/-}$+NAM to *Atg5*$^{-/-}$)	Log2 (FC)	-Log10 (P-adjusted)
**NAD^{+}**	**0.43**	**2.12**
Cis-aconitate	0.54	2.12
**NADH**	**0.53**	**1.67**
Sedoheptulose-7-phosphate	0.87	1.67
Glyceraldehyde-3-phosphate	2.14	1.57
Citrate	0.43	1.57
**UMP**	**0.27**	**1.30**
Alpha-D-glucose	0.40	1.30
2-oxoglutarate	0.13	1.27
**CMP**	**0.71**	**0.90**
Pyruvate	0.36	0.84
**ATP**	**0.22**	**0.60**

D

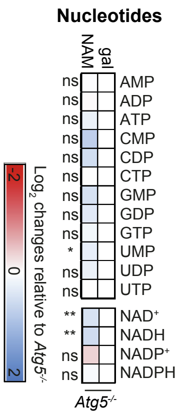

Figure S2. Interference with the NAD(H) biosynthesis in respiring cells affects cell viability and metabolism. Related to Figure 2.

A, B, Cytotoxicity assay (A) and phase-contrast images (B) in *Atg5*$^{+/+}$ MEFs cultured for 60 h (A) or 48 h (B) in glucose (glu) or galactose (gal) medium supplemented with FK866 or solvent (DMSO). **C,** List of discovered depleted metabolites in *Atg5*$^{-/-}$ MEFs treated for 16 h with gal medium in the absence or presence of 3 mM NAM. Highlighted are metabolites that change significantly ($-0.51 \geq Log_2(FC) \geq 0.49$, log10($P$ adjusted>1.3) (highlighted in red) and correlate with cell death/survival (bold). **D,** Metabolite profiling in *Atg5*$^{-/-}$ MEFs treated as in (H) is depicted as a heatmap of Log2 (fold change (FC)) of *Atg5*$^{-/-}$+NAM to *Atg5*$^{-/-}$ MEFs, showing only the nucleotides. Graphical data are mean ± s.e.m of n = 3 biological replicates (A). P values were calculated by one-way ANOVA followed by multiple comparisons with the two-stage linear step-up procedure of Benjamini, Krieger and Yekutieli (A) or multiple t-test with the original FDR method of Benjamini and Hochberg (C, D) on three independent experiments. *$P<0.05$; **$P<0.01$; ***$P<0.001$; ns (non-significant). Scale bar, 200 µm (B).

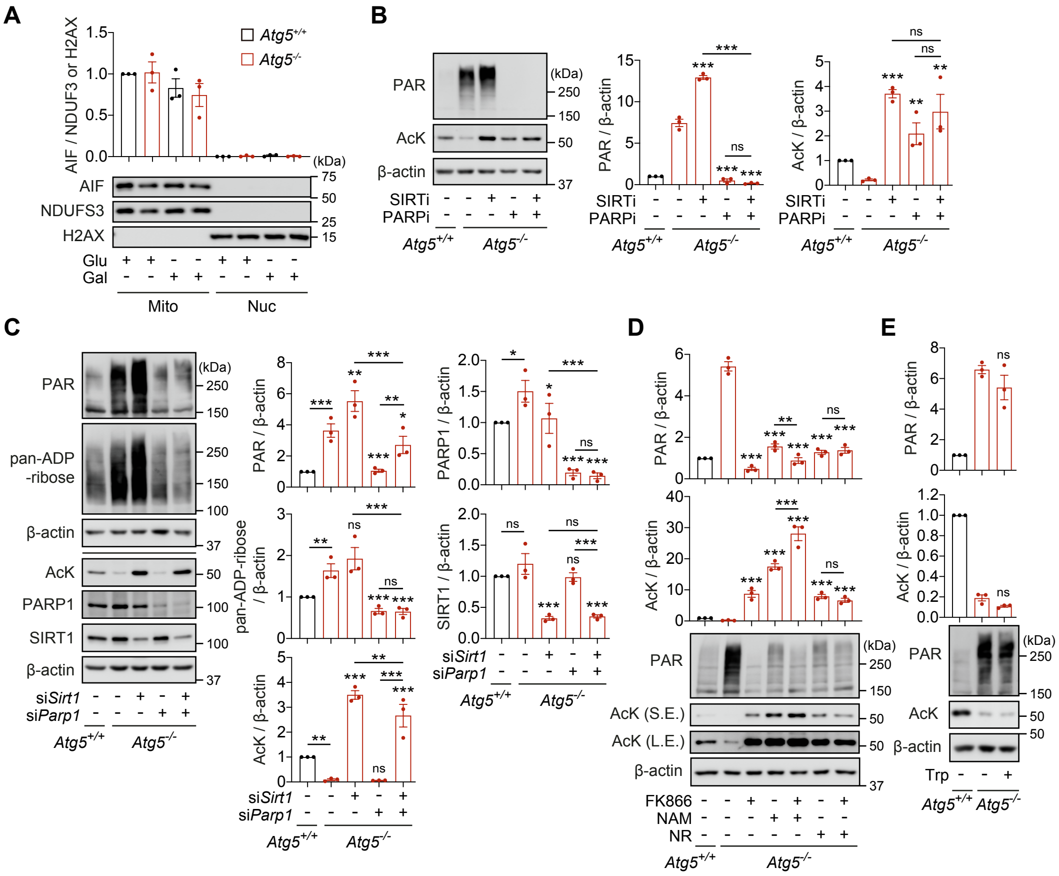
A
B
C
D
E
AIF / NDUF3 or H2AX
Atg5+/+
Atg5-/-
AIF
NDUFS3
H2AX
Glu
Gal
Mito
Nuc
PAR
AcK
β-actin
SIRTi
PARPi
PAR / β-actin
AcK / β-actin
pan-ADP -ribose
PARP1
SIRT1
siSirt1
siParp1
PARP1 / β-actin
pan-ADP-ribose / β-actin
SIRT1 / β-actin
AcK (S.E.)
AcK (L.E.)
FK866
NAM
NR
Trp
(kDa)

Figure S3. Effects of NADase inhibitors, silencing NADases and NAD(H) boosting agents on NADase activity in *Atg5*$^{-/-}$ MEFs. Related to Figures 2 and 3.

A, Immunoblot analyses of mitochondrial (Mito) and nuclear (Nuc) proteins (AIF, NDUFS3, H2AX) extracted from *Atg5*$^{+/+}$ and *Atg5*$^{-/-}$ MEFs after 24 h culture in glu or gal medium. **B,** Immunoblot analyses for PAR and AcK in *Atg5*$^{+/+}$ and *Atg5*$^{-/-}$ MEFs cultured for 14 h in gal medium supplemented with sirtinol (SIRTi), olaparib (PARPi), the combination of both drugs or solvent (DMSO). **C,** Immunoblot analyses for PAR, AcK, pan-ADP-ribose, PARP1 and SIRT1 in *Atg5*$^{+/+}$ and *Atg5*$^{-/-}$ MEFs transfected with *Parp1*, *Sirt1*, the combination of both siRNAs or *Control* siRNA and cultured in gal medium for 14 h. **D,** Immunoblot analyses of PAR and AcK in *Atg5*$^{+/+}$ and *Atg5*$^{-/-}$ MEFs cultured for 14 h in gal medium with treatment of NAM or NR in the presence or absence of FK866; S.E, short exposure; L.E. long exposure. **E,** Immunoblot analyses of PAR and AcK in *Atg5*$^{+/+}$ and *Atg5*$^{-/-}$ MEFs cultured for 14 h in gal medium with treatment of L-tryptophan (Trp). Graphical data are mean ± s.e.m of n = 3 biological replicates (A, B, C, D, E). P values were calculated by one-way ANOVA followed by multiple comparisons with the two-stage linear step-up procedure of Benjamini, Krieger and Yekutieli on three independent experiments (B, C, D, E). *P<0.05; **P<0.01; ***P<0.001; ns (non-significant).

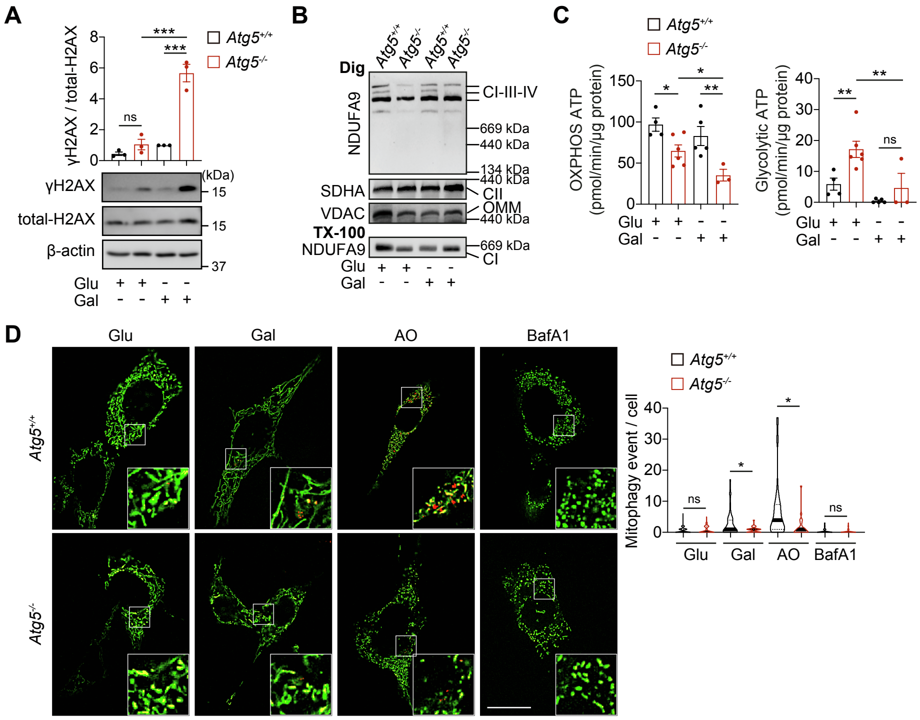
A
γH2AX / total-H2AX
Atg5+/+
Atg5-/-
ns
***
***
(kDa)
γH2AX
total-H2AX
β-actin
15
15
37
Glu + + - -
Gal - - + +
B
Dig
Atg5+/+ Atg5-/- Atg5+/+ Atg5-/-
NDUFA9
CI-III-IV
669 kDa
440 kDa
134 kDa
SDHA
CII
VDAC
OMM
TX-100
NDUFA9
CI
Glu + + - -
Gal - - + +
C
OXPHOS ATP (pmol/min/μg protein)
Glycolytic ATP (pmol/min/μg protein)
ns
D
Glu
Gal
AO
BafA1
Atg5+/+
Atg5-/-
Mitophagy event / cell
ns
ns

Figure S4. Mitochondrial and DNA damage phenotypes in *Atg5*$^{-/-}$ cells in galactose media. Related to Figure 4.

A–C, γH2AX immunoblot analyses (A), Blue native PAGE (BN-PAGE) analyses of mitochondrial complex and supercomplex assembly (extracted using digitonin (Dig) and triton-X100 (TX-100) as indicated) (B), and Seahorse analyses of ATP production from OXPHOS (left) and glycolysis (right) (C) of *Atg5*$^{+/+}$ or *Atg5*$^{-/-}$ MEFs cultured in glu or gal medium for 24 h. **D,** Fluorescence microscopy images and quantification of mitophagy of *Atg5*$^{+/+}$ or *Atg5*$^{-/-}$ MEFs expressing mt-mKeima, cultured in glu or gal medium for 24 h. Cells were treated with or without the combination of 1 μM antimycin A and 1 μM oligomycin (AO) or 400 nM bafilomycin A1 (BafA1) in glu medium for the last 4 h. Graphical data are mean ± s.e.m. of n = 3–6 (A, C) biological replicates as indicated, or displayed as violin plot (D). P values were calculated by one-way ANOVA followed by multiple comparisons with the two-stage linear step-up procedure of Benjamini, Krieger and Yekutieli (A, C, D) on three independent experiments. *P<0.05; **P<0.01; ***P<0.001; ns (non-significant). Scale bar, 20 μm (D).

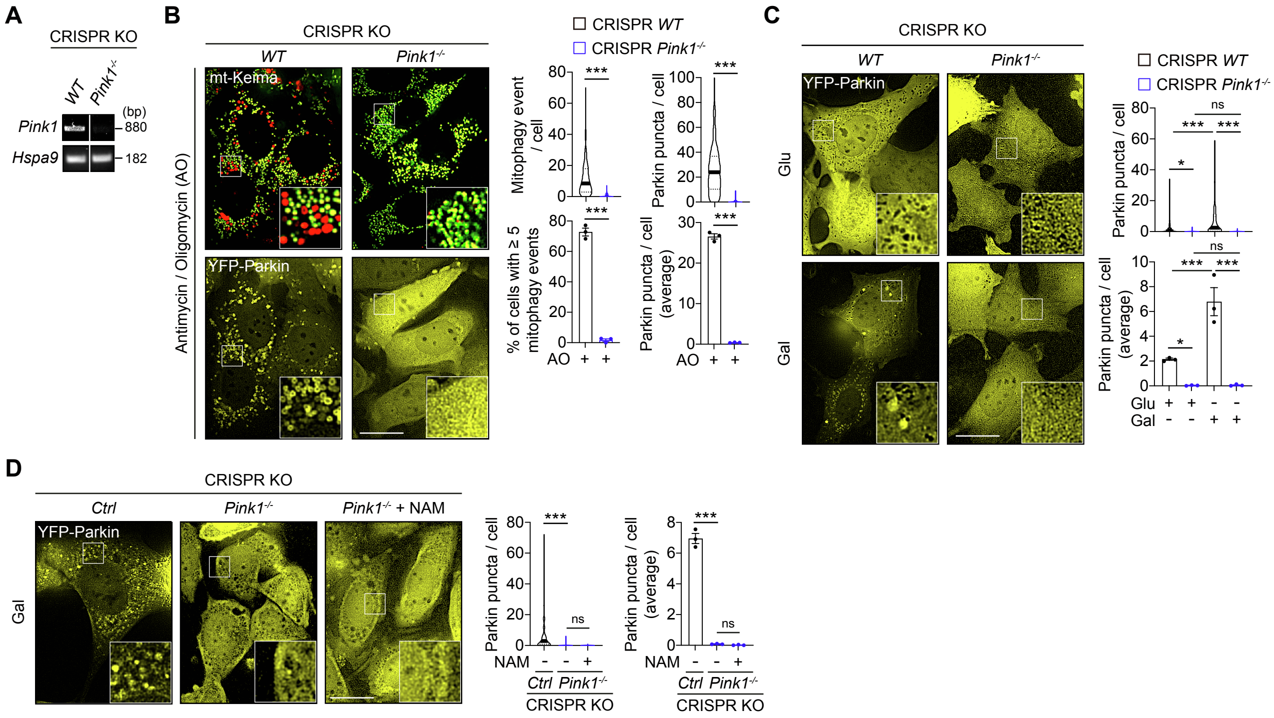
A
CRISPR KO
WT
Pink1-/-
(bp)
Pink1
880
Hspa9
182
B
CRISPR KO
WT
Pink1-/-
mt-Keima
YFP-Parkin
Antimycin / Oligomycin (AO)
CRISPR WT
CRISPR Pink1-/-
Mitophagy event / cell
Parkin puncta / cell
% of cells with ≥ 5 mitophagy events
Parkin puncta / cell (average)
AO + +
***
C
CRISPR KO
WT
Pink1-/-
YFP-Parkin
Glu
Gal
CRISPR WT
CRISPR Pink1-/-
Parkin puncta / cell
Parkin puncta / cell (average)
ns
***
*
Glu + + - -
Gal - - + +
D
CRISPR KO
Ctrl
Pink1-/-
Pink1-/- + NAM
YFP-Parkin
Gal
Parkin puncta / cell
Parkin puncta / cell (average)
NAM - - +
Ctrl Pink1-/-
CRISPR KO
***
ns

Figure S5. Loss of PINK1 suppresses the recruitment of Parkin to mitochondria. Related to Figure 5.

A, RT-PCR analyses of *Pink1* and *Hspa9* mRNA expression in isogenic non-targeted control (CRISPR *WT*) or $Pink1^{-/-}$ (CRISPR $Pink1^{-/-}$) MEFs generated by the CRISPR/Cas9 system. **B,** Fluorescence microscopy images and quantification of mitophagy and Parkin recruitment in CRISPR *WT* or $Pink1^{-/-}$ MEFs expressing mt-mKeima and YFP-Parkin, cultured in glu medium and treated with AO for 3 h. **C, D,** Fluorescence microscopy images and quantification of Parkin recruitment in CRISPR *WT* or $Pink1^{-/-}$ MEFs expressing mt-mKeima and YFP-Parkin, in the same fields and conditions as Figure 5A and F, respectively. Graphical data are mean ± s.e.m. of n = 3 (B, C, D) biological replicates, or displayed as violin plot (B, C, D). *P* values were calculated by unpaired two-tailed Student's *t*-test (B) or one-way ANOVA followed by multiple comparisons with the two-stage linear step-up procedure of Benjamini, Krieger and Yekutieli (C, D) on three independent experiments. *P<0.05; ***P<0.001; ns (non-significant). Scale bar, 20 μm (B ,C, D).

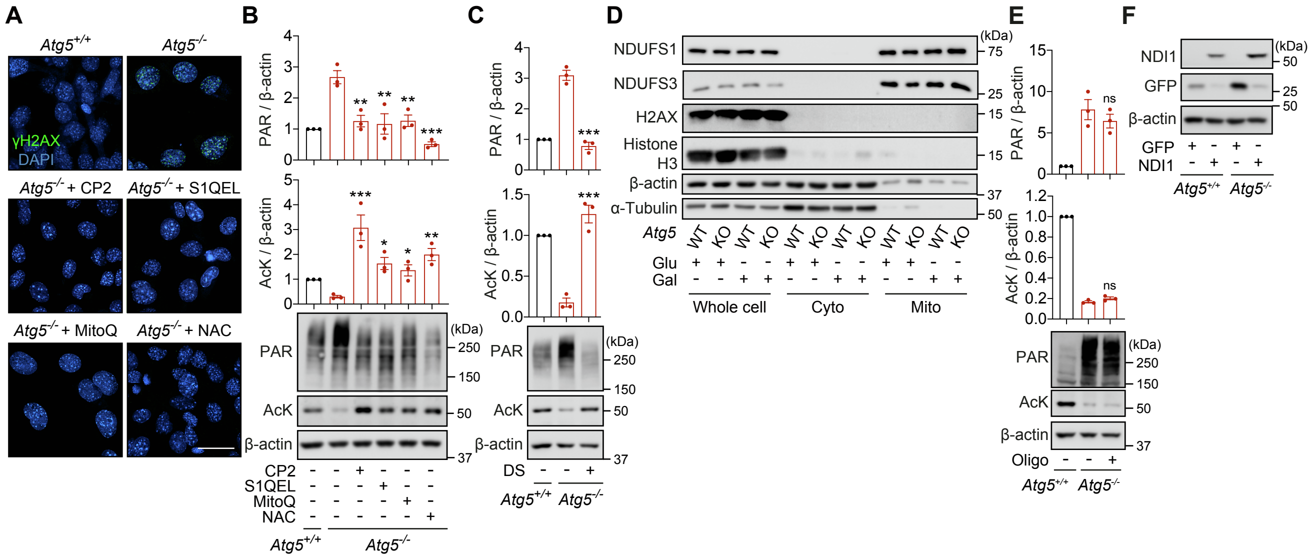

A
Atg5+/+
Atg5-/-
γH2AX
DAPI
Atg5-/- + CP2
Atg5-/- + S1QEL
Atg5-/- + MitoQ
Atg5-/- + NAC
B
PAR / β-actin
AcK / β-actin
PAR
AcK
β-actin
(kDa)
250
150
50
37
CP2
S1QEL
MitoQ
NAC
C
DS
D
NDUFS1
NDUFS3
H2AX
Histone H3
β-actin
α-Tubulin
Atg5
Glu
Gal
Whole cell
Cyto
Mito
E
Oligo
F
NDI1
GFP

Figure S6. Effects of mitochondria-targeting interventions on DNA damage and NADase activity. Related to Figures 6 and 7.

A, Immunofluorescence images (A) and immunoblot analyses for PAR and AcK (B) of *Atg5*$^{+/+}$ or *Atg5*$^{-/-}$ MEFs cultured for 14 h in gal medium supplemented with CP2, S1QEL2.2 (S1QEL), MitoQ or NAC. **C,** Immunoblot analyses for PAR and AcK of *Atg5*$^{+/+}$ or *Atg5*$^{-/-}$ MEFs cultured for 14 h in gal medium supplemented with dimethyl succinate (DS). **D,** Immunoblot analyses for mitochondrial proteins (NDUFS1, NDUFS3), nuclear proteins (H2AX, Histone H3) and cytoplasmic proteins (β-actin, α-tubulin) in whole cell lysate (Whole cell), cytoplasmic (Cyto) and mitochondrial (Mito) fractions from *Atg5*$^{+/+}$ or *Atg5*$^{-/-}$ MEFs cultured for 20 h in glu or gal medium. **E,** Immunoblot analyses for PAR and AcK of *Atg5*$^{+/+}$ or *Atg5*$^{-/-}$ MEFs cultured for 14 h in gal medium supplemented with oligomycin (Oligo). **F,** Immunoblot analyses of NDI1 and GFP in *Atg5*$^{+/+}$ and *Atg5*$^{-/-}$ MEFs expressing NDI1-IRES-GFP (NDI1) or GFP after 14 h culture in gal medium. Graphical data are mean ± s.e.m of n = 3 biological replicates (B, C, E). P values were calculated by unpaired two-tailed Student's t-test (C, E) or one-way ANOVA followed by multiple comparisons with the two-stage linear step-up procedure of Benjamini, Krieger and Yekutieli (B) on three independent experiments. *P<0.05; **P<0.01; ***P<0.001; ns (non-significant) with respect to untreated *Atg5*$^{-/-}$ MEFs. Scale bar, 20 μm (A).

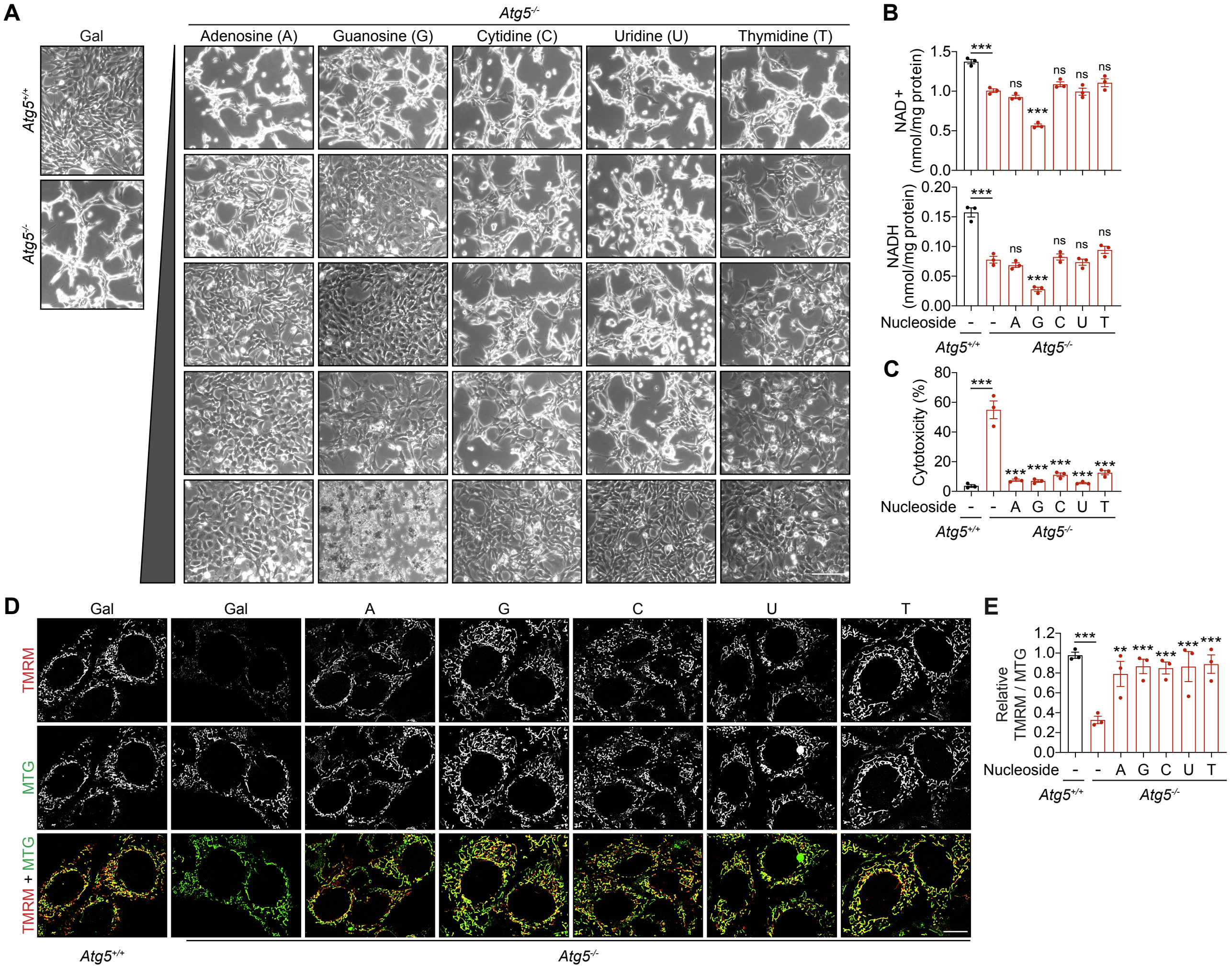
A
Gal
Atg5-/-
Adenosine (A)
Guanosine (G)
Cytidine (C)
Uridine (U)
Thymidine (T)
Atg5+/+
Atg5-/-
B
NAD+ (nmol/mg protein)
NADH (nmol/mg protein)
Nucleoside
- - A G C U T
Atg5+/+
Atg5-/-
***
ns
C
Cytotoxicity (%)
D
Gal Gal A G C U T
TMRM
MTG
TMRM + MTG
Atg5+/+
Atg5-/-
E
Relative TMRM / MTG
Nucleoside
- - A G C U T
Atg5+/+
Atg5-/-
**
***

Figure S7. Nucleoside supplementation prevents mitochondrial depolarisation and cell death by acting downstream of NAD(H) depletion in *Atg5*$^{-/-}$ MEFs. Related to Figure 7.

A, Phase-contrast images of *Atg5*$^{+/+}$ and *Atg5*$^{-/-}$ MEFs after 24 h culture in galactose (gal) medium supplemented with the different concentrations of nucleosides: 0.1, 0.3, 1, 3, 10 mM adenosine (A), cytidine (C), uridine (U), thymidine (T) or 0.05, 0.15, 0.5, 1.5, 5 mM guanosine (G). **B, C,** Measurement of NAD^+ and NADH levels (B) and cytotoxicity assay (C) in *Atg5*$^{+/+}$ and *Atg5*$^{-/-}$ MEFs cultured in gal medium supplemented with 1 mM A, 1 mM G, 10 mM C, 10 mM U or 10 mM T for 20 h (B) or 40 h (C). **D, E,** Confocal fluorescence images (D) and $\Delta\Psi m$ quantified as a relative ratio of TMRM to MTG (E) of *Atg5*$^{+/+}$ and *Atg5*$^{-/-}$ MEFs after 20 h culture in gal medium supplemented with 1 mM A, 1 mM G, 10 mM C, 10 mM U or 10 mM T and co-stained with TMRM and MTG. Graphical data are mean ± s.e.m. of $n = 3$ biological replicates (B, C, E). *P* values were calculated by one-way ANOVA followed by multiple comparisons with the two-stage linear step-up procedure of Benjamini, Krieger and Yekutieli on three independent experiments (B, C, E). *$P<0.05$; **$P<0.01$; ***$P<0.001$; ns (non-significant) with respect to untreated *Atg5*$^{-/-}$ MEFs. Scale bars, 200 µm (A); 20 µm (D).

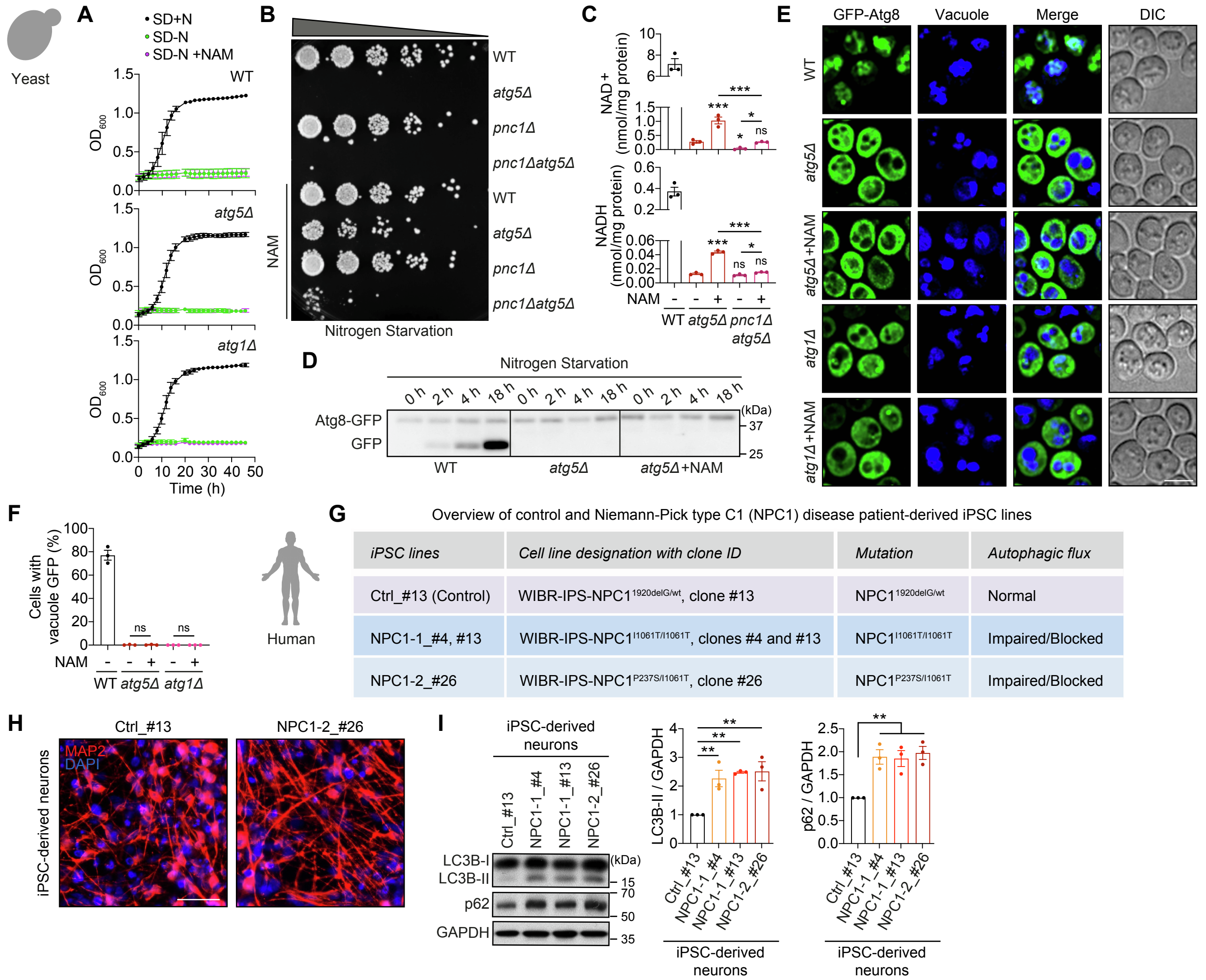

iPSC lines	Cell line designation with clone ID	Mutation	Autophagic flux
Ctrl_#13 (Control)	WIBR-IPS-NPC1$^{1920delG/wt}$, clone #13	NPC1$^{1920delG/wt}$	Normal
NPC1-1_#4, #13	WIBR-IPS-NPC1$^{I1061T/I1061T}$, clones #4 and #13	NPC1$^{I1061T/I1061T}$	Impaired/Blocked
NPC1-2_#26	WIBR-IPS-NPC1$^{P237S/I1061T}$, clone #26	NPC1$^{P237S/I1061T}$	Impaired/Blocked

Figure S8. Characterization of yeast and human neuronal models of autophagy deficiency. Related to Figure 8.

A, Growth curve analysis of BY4741 (WT), ScPPS2 (*atg5Δ*) and sSUN99 (*atg1Δ*) yeast strains cultured in nutrient-rich (SD+N) or nitrogen starvation (SD-N) media supplemented with or without 10 mM NAM. **B,** Spot-testing (5-fold serial dilutions) of S288C WT, *atg5Δ, pnc1Δ* and *pnc1Δatg5Δ* double knockout yeast strains on nutrient-rich agar media (B) and measurement of NAD^+ and NADH levels (C) of the strains following 5 days (B) or 3 days (C) of nitrogen starvation in the presence of 10 mM NAM or solvent (H_2O). **D,** Immunoblot detection of Atg8-GFP cleavage as a readout of autophagy activation in S288C WT and *atg5*Δ yeast strains at the indicated time points. Displayed immunoblot is a representative of two independent experiments. **E, F,** Fluorescence images (E) and quantification of GFP-Atg8/vacuole (stained with CMAC blue) colocalisation of sRK14 (WT), sRK15 (*atg1Δ*) and sRK16 (*atg5Δ*) yeast strains cultured for 4 h in SD-N media supplemented with 10 mM NAM. **G,** Overview of control and NPC1 patient-derived hiPSC lines, as previously described [35]. **H,** Immunofluorescence images of MAP2 in control (Con_#13) and NPC1 (NPC1-2_#26) hiPSC-derived neurons (4-weeks old). **I,** Immunoblotting analyses of LC3 and p62 in control (Con_#13) and NPC1 (NPC1-1_#4, NPC1-1_#13, NPC1-2_#26) hiPSC-derived neurons (4-weeks old); increased LC3-II and p62 levels in NPC1 neurons indicate an impairment in autophagic flux due to a block in autophagy at a late stage of the process, as previously described [17,35]. Graphical data are mean ± s.e.m. of $n = 3$ biological replicates (A, C, F, I). *P* values were calculated by unpaired two-tailed Student's *t*-test (F) or one-way ANOVA followed by multiple comparisons with the two-stage linear step-up procedure of Benjamini, Krieger and Yekutieli on three independent experiments (C, I).

*$P<0.05$; **$P<0.01$; ***$P<0.001$; ns (non-significant) with respect to untreated *atg5Δ* yeast or between the indicated groups. Scale bars, 5 μm (E); 100 μm (H).

Table S2. sgRNA sequences, related to the STAR Methods.

Target gene		Primer sequence
Atg5	forward	caccgCTTTCATCCAGAAGCTGTTC
	reverse	aaacGAACAGCTTCTGGATGAAAGc
Atg7	forward	caccgCACTGAACTCCAACGTCAAG
	reverse	aaacCTTGACGTTGGAGTTCAGTGc
Rb1cc1	forward	caccgCTAACAGCTCTATTACAAGG
	reverse	aaacCCTTGTAATAGAGCTGTTAGc
Pink1	forward	caccgCATGGTGGCTTCATACACAG
	reverse	aaacCTGTGTATGAAGCCACCATGc

Table S3. siRNAs, related to the STAR Methods.

**Target gene**	**Horizon Discovery Cat #**
Non-targeting *control*	D-001810-10
Sirt1	L-049440-00-0005
Parp1	L-040023-00-0005

Table S4. Primers for RT-PCR, related to the STAR Methods.

Target gene		Primer sequence
Pink1	forward	GCCCAGGCCAGGTCGTAA
	reverse	TCGCACACTGTTCCTCGTTA
Hspa9	forward	TGCCTCCAATGGTGATGCTT
	reverse	CAGCATCCTTAGTGGCCTGT
